# Glycosylated Porphyra-334 and Palythine-Threonine from the Terrestrial Cyanobacterium *Nostoc commune*

**DOI:** 10.3390/md11093124

**Published:** 2013-08-26

**Authors:** Ehsan Nazifi, Naoki Wada, Minami Yamaba, Tomoya Asano, Takumi Nishiuchi, Seiichi Matsugo, Toshio Sakamoto

**Affiliations:** 1 Division of Life Science, Graduate School of Natural Science and Technology, Kanazawa University, Kakuma, Kanazawa 920-1192, Japan; E-Mail: nazifiehsan@yahoo.com; 2 School of Natural System, College of Science and Engineering, Kanazawa University, Kakuma, Kanazawa 920-1192, Japan; E-Mails: naoki-wada@se.kanazawa-u.ac.jp (N.W.); s-matsugoh@se.kanazawa-u.ac.jp (S.M.); 3 Division of Biological Sciences, Graduate School of Natural Science and Technology, Kanazawa University, Kakuma, Kanazawa 920-1192, Japan; E-Mail: daba@stu.kanazawa-u.ac.jp; 4 Division of Functional Genomics, Advanced Science Research Center, Kanazawa University, Takara, Kanazawa 920-0934, Japan; E-Mails: asano@staff.kanazawa-u.ac.jp (T.A.); tnish9@staff.kanazawa-u.ac.jp (T.N.)

**Keywords:** anhydrobiosis, antioxidant, mycosporine-like amino acid (MAA), *Nostoc commune*, UV protectant

## Abstract

Mycosporine-like amino acids (MAAs) are water-soluble UV-absorbing pigments, and structurally different MAAs have been identified in eukaryotic algae and cyanobacteria. In this study novel glycosylated MAAs were found in the terrestrial cyanobacterium *Nostoc commune* (*N. commune*). An MAA with an absorption maximum at 334 nm was identified as a hexose-bound porphyra-334 derivative with a molecular mass of 508 Da. Another MAA with an absorption maximum at 322 nm was identified as a two hexose-bound palythine-threonine derivative with a molecular mass of 612 Da. These purified MAAs have radical scavenging activities *in vitro*, which suggests multifunctional roles as sunscreens and antioxidants. The 612-Da MAA accounted for approximately 60% of the total MAAs and contributed approximately 20% of the total radical scavenging activities in a water extract, indicating that it is the major water-soluble UV-protectant and radical scavenger component. The hexose-bound porphyra-334 derivative and the glycosylated palythine-threonine derivatives were found in a specific genotype of *N. commune*, suggesting that glycosylated MAA patterns could be a chemotaxonomic marker for the characterization of the morphologically indistinguishable *N. commune*. The glycosylation of porphyra-334 and palythine-threonine in *N. commune* suggests a unique adaptation for terrestrial environments that are drastically fluctuating in comparison to stable aquatic environments.

## 1. Introduction

The terrestrial cyanobacterium *Nostoc commune* (*N. commune*) is a cosmopolitan species that is distributed from the tropics to the polar regions of the Earth [1]. *N. commune* adapts to terrestrial environmental conditions [1,2]. In its natural habitats, it forms visually conspicuous colonies that are subjected to frequent cycles of desiccation and wetting. Desiccated colonies have no metabolic activity and retain the ability to grow for more than 100 years [3,4]. Upon rehydration, *N. commune* cells rapidly recover respiration and photosynthesis [5,6,7,8]. This phenomenon is termed anhydrobiosis [9,10,11,12]. *N. commune* is considered to be a prokaryotic model anhydrobiote that retains oxygenic photosynthetic capabilities in vegetative cells and does not differentiate into akinetes (spores) [1,12]. In addition to extreme desiccation tolerance, *N. commune* colonies are exposed to direct solar radiation and can tolerate UV radiation stress [13,14]. The mechanisms behind this extreme environmental stress tolerance are thought to involve multiple processes, and the ability to produce a biochemically complex extracellular matrix appears to be a required factor [15]. The structural constituents of this matrix in *N. commune* include extracellular polysaccharides (EPS) [15], water stress protein (WspA) [16] and UV absorbing pigments [14]. EPS, which account for 80% of the dry weight of *N. commune* colonies [17], play a major role in mechanisms, including desiccation tolerance, that protect cells from various stresses in severe habitats [7,15]. 

Mycosporine-like amino acids (MAAs) are water-soluble and absorb specific UV-B radiation in the range of 280–320 nm [18,19,20,21,22,23,24]. Structurally distinct MAAs have been observed in eukaryotic algae and cyanobacteria, including marine, freshwater and terrestrial species [18,19,20]. MAAs have an important role in the overall strategy to reduce the deleterious effects of desiccation and environmental UV radiation [25], especially for the adaptation to terrestrial environments exposed to higher levels of UV-radiation than aqueous environments [26]. With their photoprotective and antioxidative properties, MAAs are natural bioactive compounds attractive to cosmeceutical and pharmaceutical applications [19,20]. Physiological responses to osmotic water stress induced by high salt concentrations are thought to overlap with responses to matric water stress in dry environments [27]. It has been suggested that MAAs may function as osmotic solutes because of the accumulation of MAAs in halophilic cyanobacteria [28] and overlap between anti-stress compounds produced by marine and terrestrial cyanobacteria can be expected. 

*N. commune* synthesizes UV-A/B absorbing compounds that are secreted to the extracellular matrix [14,15,16]. Recently, two novel glycosylated MAAs were found in naturally growing *N. commune* colonies [29]. An MAA with an absorption maximum at 335 nm and a molecular mass of 478 Da was identified as a pentose-bound porphyra-334 derivative. The other identified MAA had double absorption maxima at 312 and 340 nm and a molecular mass of 1050 Da. The 544-Da lipid-soluble pigment scytonemin absorbs UV-A radiation of 320–400 nm and occurs exclusively in cyanobacterial sheaths [13,14]. These glycosylated MAAs [29] and scytonemin [30,31] have radical scavenging activities *in vitro*. Additionally, a unique antioxidative compound, nostocionone, was reported in *N. commune* [31]. *N. commune* is considered to adapt to terrestrial environments with high levels of solar radiation, as it produces both glycosylated MAAs and scytonemin with antioxidative activities in its extracellular matrix [14,15,16,29,30,32].

*N. commune* is known to be genetically diverse, and four major genotypes, which are hardly morphologically distinguishable and have genetic differences that are not great enough to be recognized as distinct species, have been reported in Japan. These genotypes have been observed within small areas, such as the Kakuma Campus of Kanazawa University [33]. During the investigation of MAAs in naturally growing colonies from different sampling locations, we found novel MAAs that were neither the 478-Da MAA nor the 1050-Da MAA. In this study, we purified and characterized the chemical structures and radical scavenging activities of these newly found glycosylated MAAs. These findings provide new insights into the diversity and chemotaxonomic features of MAAs, as well as the biological functions of MAAs in the adaptation of the cyanobacterium *N. commune* to terrestrial environments.

## 2. Results and Discussion

### 2.1. UV Absorption Spectra of Water Extracts and Genotypes of *N. commune*

**Figure 1:**
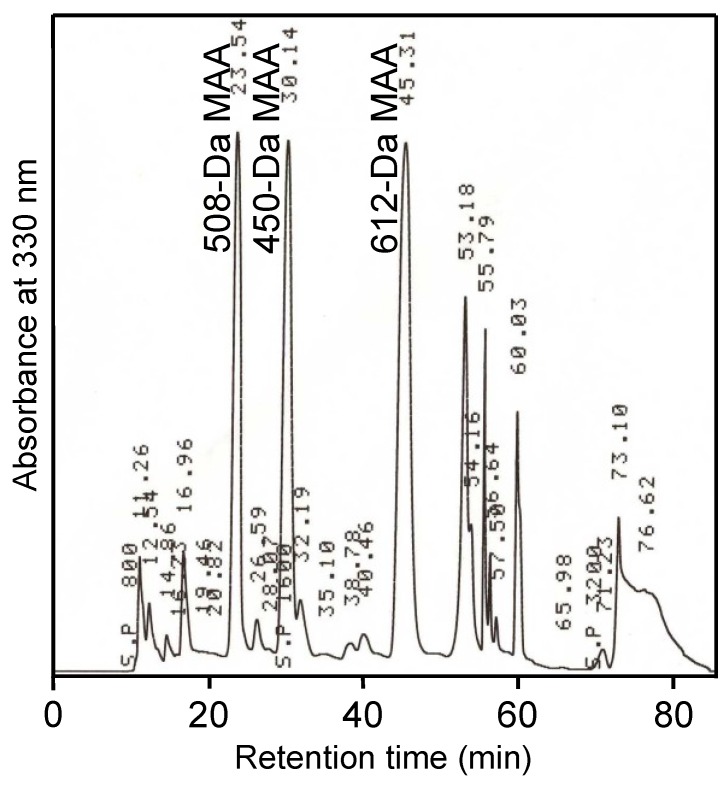
HPLC chromatogram of a water extract of *N. commune* (genotype D). The water extract was injected into an HPLC system equipped with a reverse phase column (IRICA C18, 20 × 250 mm). The mobile phase changed in a stepwise fashion from distilled water for the initial 40 min, to 0.1% acetic acid 10% methanol for the next 20 min and to 100% methanol for the final 20 min. The flow rate was constant at 4 mL·min^−1^, and the A_330_ was monitored. Samples of 508-Da mycosporine-like amino acid (MAA) with an absorption maximum at 334 nm and 450-Da MAA and 612-Da MAA with an absorption maximum at 322 nm were eluted at 23, 30 and 45 min, respectively.

*N. commune* colonies were found for which the water extract showed a characteristic UV-absorbing spectrum with an absorption maximum at 325 nm. However, this spectrum was different from known *N. commune* spectra, and thus, the MAA profile and genotype of this particular *N. commune* sample were examined further. In a typical HPLC chromatogram of the water extract with an absorption maximum at 325 nm, three distinct MAAs were detected as major MAAs with different retention times (Figure 1). Because the retention times and UV absorption maxima were different from those of other known MAAs from *N. commune*, these MAAs were purified and characterized as described below. According to an analysis of the 16S rRNA gene sequence, the *N. commune* colony that contained MAAs with absorption maxima at approximately 325 nm was identified as genotype D, as described by Arima *et al.* [33]. 

### 2.2. 508-Da MAA with an Absorption Maximum at 334 nm

**Figure 2:**
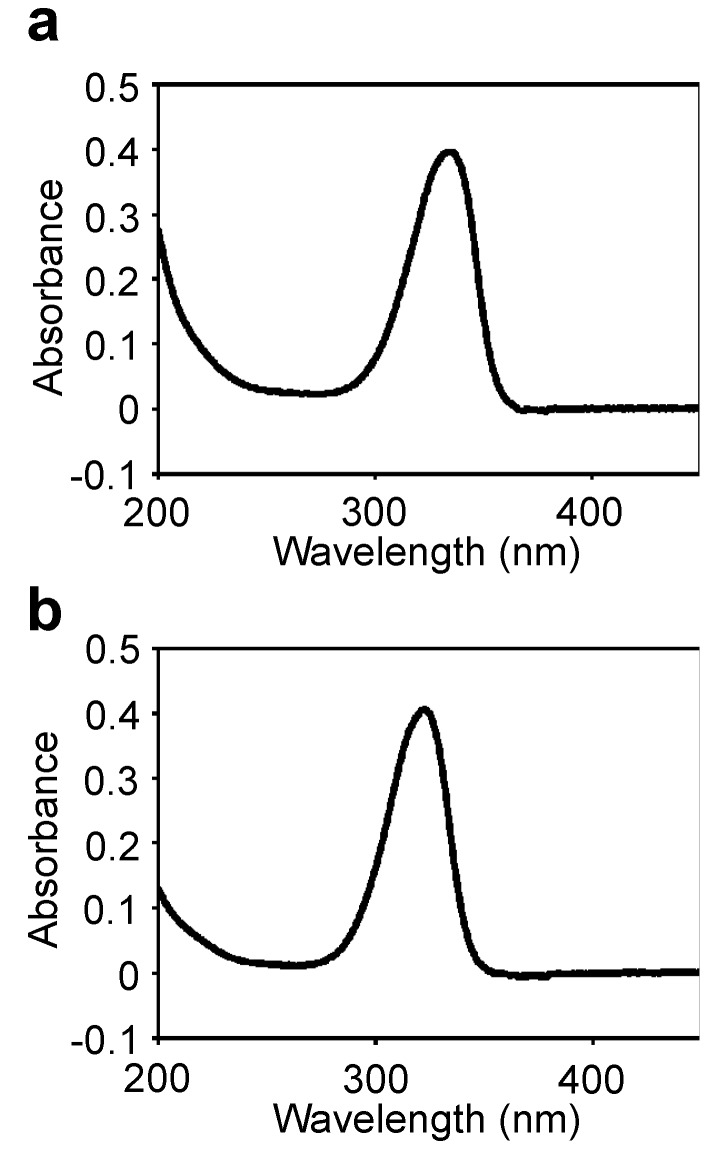
UV absorption spectra of the purified MAAs in H_2_O. (**a**) 508-Da MAA with absorption maxima at 334 nm (ε = 3.63 × 10^4^ M^−1^·cm^−1^ at 334 nm); (**b**) 612-Da MAA with absorption maximum at 322 nm (ε = 2.82 × 10^4^ M^−1^·cm^−1^ at 322 nm).

A MAA with an absorption maximum at 334 nm (Figure 1) was purified from the field-isolated natural *N. commune* colonies (Table 1, Figure S1). The UV absorption spectrum of the purified MAA showed a single absorption peak at 334 nm (Figure 2a). The absorption maximum shifted reversibly to 332 nm in a high acidic solution (pH < 2) and to 334 nm in alkaline solutions. The molecular mass was 508 Da, according to MALDI-TOF MS (Table 2). The absorption coefficient of this MAA in water was 71.4 L·g^−1^·cm^−1^ at 334 nm and the calculated molar absorption coefficient at 334 nm was 3.63 × 10^4^ M^−1^·cm^−1^. Because no MAA with a molecular mass of 508 Da had been previously reported, the chemical structure of the 508 Da MAA was further characterized. 

**Table 1:** Purification of 508-Da, 450-Da and 612-Da mycosporine-like amino acids (MAAs) with absorption maxima at 334, 322 and 322 nm, respectively, from *N. commune* ^a^*.*

Step		Volume (mL)	MAA concentration ^b^ (mg·L^−1^)	MAA amount (mg)	Yield (%)
Water extract		640	19.4	12.4	100
70% Ethanol solution		1960	5.4	10.6	85
Vacuum concentration		3.5	2374	8.3	67
Reverse-phase HPLC	508-Da	0.5	3525	1.8	15
	450-Da	0.5	1969	1.0	8
	612-Da	0.5	2404	1.2	10
Gel filtration	508-Da	0.5	2474	1.2	10
	450-Da	0.5	1782	0.9	7
	612-Da	0.5	2226	1.1	9

^a^ Dry colonies of *N. commune* (30 g) that contained the MAAs with absorption maxima at 334 and 322 nm were used as starting materials and the MAAs were purified as described in Experimental Section. HPLC chromatograms and absorption spectra of the purified MAAs are shown in Figure S1 in Supporting Information; ^b^ MAA concentrations were determined with an absorption coefficient of 120 L·g^−1^·cm^−1^ [34].

**Table 2:** Summary of MALDI-TOF MS analysis of the purified 508-Da MAA with absorption maximum at 334 nm.

	Mass of fragment	Relative abundance	Neutral loss	Deleted fragment
MS of purified MAA	509.12	100		
	465.13	81	44	CO_2_
	451.12	10	58	CO_2_ + CH_2_
	427.07	27	82	-
	425.04	58	84	-
	421.15	58	88	2CO_2_
	419.15	24	90	CO_2_ + 2CH_2_ + H_2_O
	407.10	40	102	2CO_2_ + CH_2_
	403.13	37	106	2CO_2_ + H_2_O
	387.11	57	122	-
	385.07	35	124	2CO_2_ + 2H_2_O
MS^2^ of *m*/*z* 509	**509.12**			
	347.27	100	162	Hexose
	303.24	6	206	Hexose + CO_2_ (or C_2_H_4_O)
	279.20	7	230	-
MS^2^ of *m*/*z* 465	**465.13**			
	303.29	100	162	Hexose
	285.27	3	180	Hexose + H_2_O
	235.22	4	230	-

**Figure 3:**
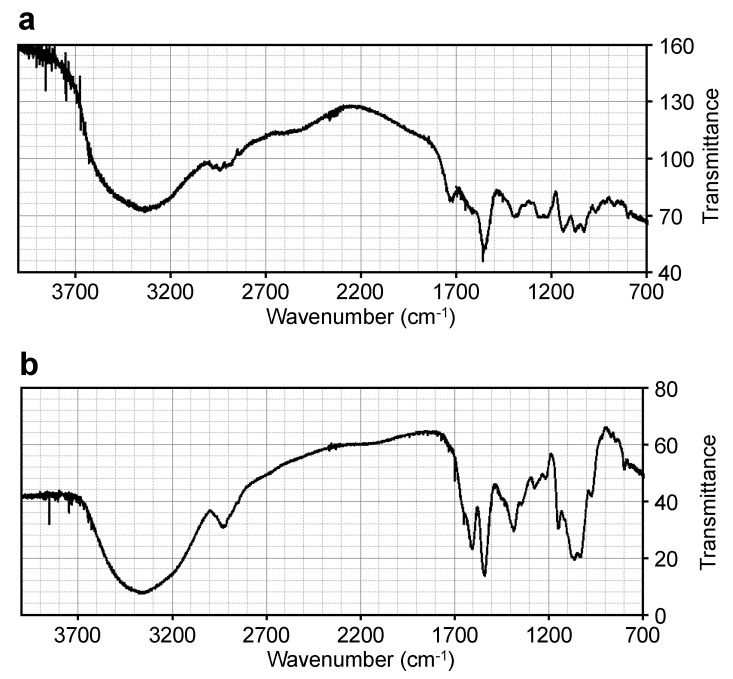
FT-IR spectra of (**a**) the 508-Da MAA with an absorption maximum at 334 nm and (**b**) the 612-Da MAA with an absorption maximum at 322 nm.

The IR spectrum of the 508-Da MAA (Figure 3a) was compared to those of known MAAs, including porphyra-334 [35] and a glycosylated porphyra-334 with a molecular mass of 478 Da from *N. commune* [29] (Table 3). Similar IR absorption peaks were observed, which suggested that the 508-Da MAA had a similar structure to the known MAAs. The absorption peak at 1560 cm^−1^ in the 508-Da MAA indicated the presence of the conjugated imine as a characteristic structure of MAA chromophore, corresponding to those at 1540 cm^−1^ in porphyra-334 and at 1558 cm^−1^ in the 478-Da glycosylated porphyra-334 derivative. The characteristic absorption peak at 3334 cm^−1^ in the 508-Da MAA indicated the presence of several hydroxyl functional groups, and the absorption peaks in the range of 1200–1300 cm^−1^ suggested the presence of a sugar, which was consistent with the predicted structure as described below. 

MALDI-TOF MS/MS analysis was performed on the parent molecular ion fragment with *m*/*z* 509 and molecular ion fragment with *m*/*z* 465 (Table 2). A fragment with *m*/*z* 347, a molecular mass identical to that of porphyra-334 [36,37], was detected in the second MS of the fragment with *m*/*z* 509. The neutral loss of 162 Da suggested the deletion of a hexose (C_6_H_10_O_5_) from the fragment with *m*/*z* 509. The fragment with *m*/*z* 303 suggested the deletion of CO_2_. The fragment with *m*/*z* 303 was also detected in the second MS of the fragment with *m*/*z* 465. The deletion of 162 Da from the fragment with *m*/*z* 465 to yield *m*/*z* 303 indicated the presence of a hexose in the MAA structure (Table 2). Additionally, in the MS analysis the fragments with *m*/*z* 465 and *m*/*z* 421 suggested the consecutive deletion of CO_2_ and the presence of two carboxyl groups in the MAA structure (Table 2). These fragmentation patterns were similar to those from the MS analyses of known MAAs [38]. These data suggest that the 508-Da MAA is a hexose-bound porphyra-334 derivative. 

**Table 3:** Comparison of the IR spectra of the purified MAAs with that of porphyra-334.

508-Da MAA with A_max_ at 334 nm	Porphyra-334	478-Da MAA with A_max_ at 335 nm
Wavenumber (cm^−1^)		
		3400
3334		
	3300	
2942, 2975		
1718		
	1600	1606
1560	1540	1558
1379, 1395	1380	1382
1301, 1355		1310
1231, 1257		1273
1138		1132
1075	1080	1072
1030		1006
968		

IR spectra were recorded with a Fourier transform infrared (FT-IR) spectrometer (Nicolet NEXUS 470 FT-IR) according to the KBr disk method. The wavenumbers of the absorption bands for the 508-Da MAA are compared to those for the known MAAs porphyra-334 [35] and 478-Da pentose-bound porphyra-334 [29]. The FT-IR spectrum of the 508-Da MAA is shown in Figure 3a.

**Figure 4:**
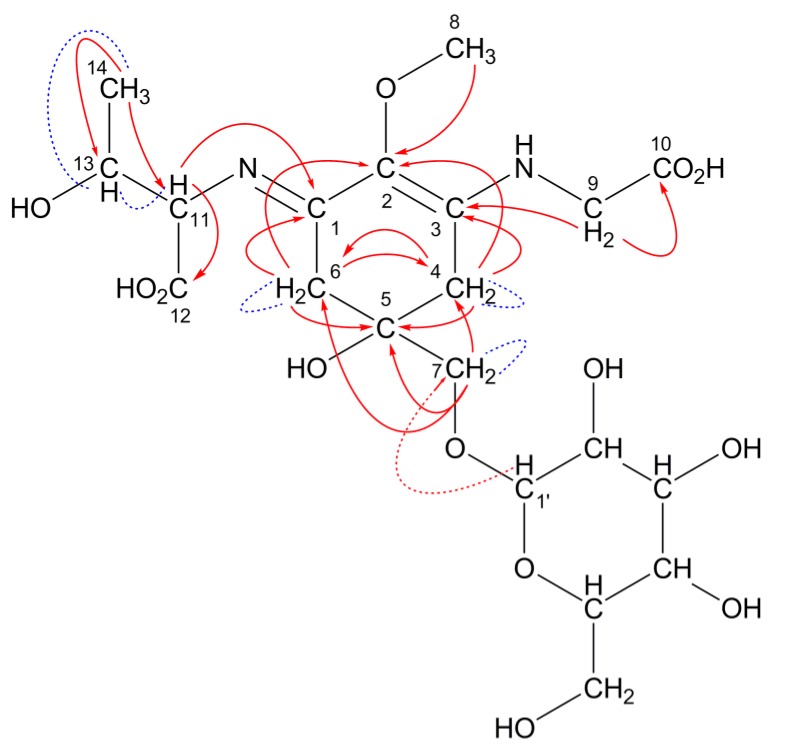
A predicted structure for the 508-Da MAA with an absorption maximum at 334 nm and a molecular formula of C_20_H_32_N_2_O_13_. Hexose is bound to porphyra-334 at C7 position. Red arrows and blue dashed lines represent the apparent HMBC and COSY correlations, respectively. The red dashed arrow represents probable HMBC correlation.

This predicted structure was confirmed by NMR spectroscopic analysis. Both the known chemical shifts for porphyra-334 and hexose were observed in the 508-Da MAA (Table 4). Typical ^13^C-chemical shifts assignable to a cyclohexenimine chromophore (C1, 2, 3, 4, 5, 6) and amino acid substituents (C9, 10, 11, 12, 13, 14) of the 508-Da MAA were observed at the same regions in porphyra-334. The characteristic coupling pattern (AB quartet) of methylene protons at C4 and C6 was determined (*J* = 17.4 Hz), which is not shown in porphyra-334. It was hard to identify which carbon (C4 and C6) should be assigned to the glycine-substituted side and *vice versa* because chemical shifts at C9 and C11 protons were very close to recognize the correlation in heteronuclear multiple bond correlation (HMBC) spectrum. However, the ^13^C-chemical shifts in the 508-Da MAA were highly similar to those in the pentose-bound porphyra-334 derivative, which has been reported previously (Table 4). The correlations in COSY and HMBC spectra are summarized in Table 5 and shown in Figure 4 as red arrows and blue dashed lines, respectively. Characteristic correlations assignable to the MAA backbone appeared and a probable HMBC correlation suggesting a putative hexose-binding site was observed (shown as red dashed arrow in Figure 4). 

**Table 4:** Summary of the NMR analysis of the 508-Da MAA with an absorption maximum at 334 nm.

C	508-Da MAA with A_max_ at 334 nm		478-Da pentose-bound porphyra-334		Porphyra-334	
	^13^C	^1^H	^13^C	^1^H	^13^C	^1^H
1 ^a^	161.4	-	161.6	-	161.6	-
2	128.6	-	128.4	-	126.0	-
3^ a′^	163.4	-	163.2	-	163.2	-
4^ b^	36.1	2.82, 3.03 (ABq, 17.4)	36.4	3.00	32.5	2.75
5	73.3	-	73.1	-	71.3	-
6^ b′^	36.6	2.80, 2.91 (ABq, 17.4)	35.9	2.83	33.0	2.77
7	76.1	3.47, 3.76 (d, 10.1, each)	77.7	3.90, 3.67	67.1	3.61 (s)
8	62.3	3.67 (s)	62.2	3.70 (s)	59.0	3.73 (s)
9	49.6	4.09 (d, 1.8)	49.5	4.06 (d, 2.4)	47.0	4.07 (s)
10^ c^	177.6	-	177.7	-	177.6	-
11	67.5	4.10 (d, 4.6)	67.3	4.09 (d, 4.5)	64.0	4.12 (d, 5.0)
12^ c′^	178.2	-	178.2	-	178.0	-
13	74.4	4.34 (dq, 4.6, 6.4)	71.0	4.31 (dq, 4.5, 6.5)	68.0	4.33 (m, 5.0, 6.4)
14	22.4	1.24 (d, 6.4)	22.3	1.26 (d, 6.5)	19.0	1.26 (d, 6.4)
					Methyl α- d-Man	
					^13^C	^1^H
1′	101.8	4.96 (d, 3.2)	106.4	4.37 (d, 7.6)	102.2	4.66
2′	71.4	3.88	73.5	3.60 (dd, 7.6, 9.6)	71.4	3.82
3′	72.2	3.97	75.0	3.68 (dd, 9.6, 3.6)	72.1	3.65
4′	70.9	3.82	71.1	3.95 (m)	68.3	3.53
5′	72.3	3.80	69.1	3.92, 3.65	73.9	3.51
6′	64.2	3.55, 3.72			62.5	3.79, 3.65

NMR spectra were recorded with a JEOL ECS400 spectrometer in D_2_O as a solvent. Chemical shifts (ppm) for the purified 508-Da MAA with A_max_ at 334 nm are compared with those for the known MAA 478-Da pentose-bound porphyra-334 [29], porphyra-334 [35] and methyl α-d-Man [39]. The coupling patterns and constants (Hz) are shown in parentheses. x, x′, Chemical shifts may be exchangeable for the 508-Da MAA.

**Table 5:** Characteristic correlations in COSY and HMBC spectra assignable to the 508-Da MAA backbone.

	COSY	HMBC
H4	H4	C2, C3, C5, C6
H6	H6	C1, C2, C4, C5
H7	H7	C4, C5, C6
H8		C2
H9		C3, C10
H11	H13	C1, C12
H13	H11, H14	
H14	H13	C11, C13
H1′		C7 ^a^

^a^ Probable correlation.

The presence of a hexose moiety in the 508-Da MAA was confirmed by the ^13^C chemical shift in methyl α-d-mannose; chemical shift at the anomer carbon (C1′) was almost the same as that in methyl α-d-mannose, indicating that the substitution position is at the anomer position. Remaining peaks were assigned to one CH_2_ (C6′) and four CH carbon (C2′, 3′, 4′, 5′) from the result of Distortionless Enhancement by Polarization Transfer (DEPT) spectrum. The chemical shifts of these peaks were also consistent with those of methyl α-d-mannose. In the 508-Da MAA, the hexose binding position could be thought of as at C7 position because the CH_2_ protons at C7 position coupled to each other (*J* = 10.1 Hz). This coupling pattern was not observed in porphyra-334 but was observed in the pentose-bound 478-Da MAA (Table 4). In our previous report, the pentose-binding position at the C7 methylene was identified because of the apparent correlation between the C7 carbon and the anomer (C1′) proton in the HMBC spectrum [29]. In the 508-Da MAA, the correlation between C7 and H1′ was not observed apparently (Table 5, Figure 4 red dashed arrow), but geminal coupling at C7 protons suggesting magnetic anisotropy was observed. Thus the high rotation barrier around C5–C7 bond due to a large substituent can be predicted and this idea agrees with the presence of a monosaccharide at C7 position of the 508-Da MAA. The dihedral angle dependence of C5–C7 rotational energy was calculated by using Chem-3D software. The rotational barrier of the C7 substituted porphyra-334 was much greater than that of porphyra-334, which cannot be surmounted at room temperature (data not shown). This simulation can be supported by the previous report concerning neocedumoside derivatives who have a similar chemical structure around a hexose-binding site [40]. Table 6 shows the chemical shifts of the hexose-binding sites of the 508-Da MAA, porphyra-334, neocedumoside and its hydrolysate. The geminal coupling of magnetically anisotropic methylene protons (*J* = 10.1 Hz) was detected due to the deshielding effect of the neighboring σ-electron. The equatorial proton was observed at lower magnetic field region by 0.43 ppm rather than the axial proton. The ^1^H-chemical shifts at C7 position in the 508-Da MAA showed two apparently separated signals by 0.29 ppm with geminal coupling between them. The hydrolysis of neocedumoside results in axial and equatorial peaks getting closer to their center of balance [40]. The center of balance of two separated signals in the 508-Da MAA was 3.61 ppm, which is identical to the corresponding chemical shift in porphyra-334. These data support the idea that magnetic anisotropy of methylene protons is caused by the substitution of hexose. In addition, the ^13^C chemical shift in neocedumoside is downfield shifted by 8.2 ppm from that of hydrolysate, suggesting a characteristic NMR feature on glycolysis. A similar shift (9.0 ppm) from porphyra-334 was also observed in the 508-Da MAA. These NMR analyses and molecular simulation support the substitution of hexose at C7 position. Figure 4 shows the predicted structure of the 508-Da MAA from *N. commune*. 

**Table 6:** Comparison of ^13^C and ^1^H chemical shifts at the hexose binding site of the 508-Da MAA, porphyra-334, neocedumoside and its hydrolysate.

	508-Da MAA	Porphyra-334	Neocedumoside	Neocedumoside-hydrolysate
^13^C	76.1	67.1	79.0	70.8
^1^H	3.47 (d, 10.1) 3.76 (d, 10.1)	3.61 (s)	3.40 (d, 10.1) 3.83 (d, 10.1)	3.51 (d, 11.0) 3.54 (d, 11.0)

Chemical shifts (ppm) for the purified 508-Da MAA are compared with those for the known MAA porphyra-334 [29], neocedumoside and its hydrolysate [40]. The coupling patterns and constants (Hz) are shown in parentheses.

### 2.3. 612-Da MAA with an Absorption Maximum at 322 nm

An MAA with an absorption maximum at 322 nm (Figure 1) was purified from the same *N. commune* colonies (Table 1, Figure S1). The UV absorption spectrum of the purified MAA showed a single absorption peak at 322 nm (Figure 2b); similar to the 508-Da MAA, the absorption maximum reversibly shifted to 320 nm in a highly acidic solution. The molecular mass was 612 Da, according to MALDI-TOF MS (Table 7). The accurate molecular mass was determined by FAB MS to predict the elemental composition. A molecular ion fragment with *m*/*z* 613.2462 was detected and its predicted molecular formula was C_24_H_41_N_2_O_16_ within 1 ppm error. The absorption coefficient of this MAA in water was 46.07 L·g^−1^·cm^−1^ at 322 nm, and its calculated molar absorption coefficient at 322 nm was 2.82 × 10^4^ M^−1^·cm^−1^. Because no MAA with a molecular mass of 612 Da had been previously reported, the chemical structure of the 612 Da MAA was further characterized. 

**Table 7:** Summary of MALDI-TOF MS analysis of the purified 612-Da MAA with absorption maximum at 322 nm.

	Mass of fragment	Relative abundance	Neutral loss	Deleted fragment
MS of purified MAA	613.17	18		
	569.19	95	44	CO_2_
	451.12	4	162	Hexose
	407.13	17	206	CO_2_ + Hexose
	389.12	100	224	CO_2_ + Hexose + H_2_O
	349.09	21	264	Hexose + C_4_H_6_O_3_
	227.02	16	386	CO_2_ + 2Hexose + H_2_O
MS^2^ of *m*/*z* 613	**613.17**			
	451.21	100	162	Hexose
	407.21	1	206	Hexose + CO_2_
	389.23	8	224	Hexose + CO_2_ + H_2_O
	317.20	6	296	-
	289.18	14	324	2Hexose
	241.17	10	372	-
	185.14	6	428	-
	91.10	6	522	-
MS^2^ of *m*/*z* 569	**569.19**			
	407.37	100	162	Hexose
	392.32	13	177	Hexose + CH_3_
	389.33	37	180	Hexose + H_2_O
	375.31	10	194	Hexose + H_2_O + CH_2_
	245.25	26	324	2Hexose
	227.24	16	342	2Hexose + H_2_O
	209.22	21	360	2Hexose + 2H_2_O
	191.21	8	378	2Hexose + 3H_2_O
	177.19	18	392	2Hexose + 3H_2_O + CH_2_
	91.10	12	478	-
MS^2^ of *m*/*z* 451	**451.21**			
	407.00	2	44	CO_2_
	289.20	100	162	Hexose
	245.17	6	206	Hexose + CO_2_
	227.19	2	224	Hexose + CO_2_ + H_2_O
	170.13	1	281	Hexose + CO_2_ + H_2_O + C_3_H_5_O
	151.14	1	300	Hexose + CO_2_ + 2H_2_O + C_3_H_6_O
	91.10	2	360	-
MS^2^ of *m*/*z* 407	**407.13**			
	245.28	100	162	Hexose
	230.24	3	177	Hexose + CH_3_
	227.25	15	180	Hexose + H_2_O
	209.23	5	198	Hexose + 2H_2_O
	199.21	4	208	Hexose + C_2_H_6_O
	177.21	6	230	Hexose + 3H_2_O + CH_2_
	91.11	3	316	-
MS^2^ of *m*/*z* 389	**389.12**			
	227.29	100	162	Hexose
	212.24	9	177	Hexose + CH_3_
	209.26	36	180	Hexose + H_2_O
	191.24	54	198	Hexose + 2H_2_O
	181.21	17	208	Hexose + C_2_H_6_O
	170.19	8	219	Hexose + C_3_H_5_O
	159.20	9	230	-
	91.11	12	298	-
MS^2^ of *m*/*z* 349	**349.09**			
	187.19	100	162	Hexose
	169.18	8	180	Hexose + H_2_O
	151.16	25	198	Hexose + 2H_2_O

Similar IR absorption peaks were observed when the 612-Da MAA IR spectrum (Figure 3b) was compared with those from the known MAA palythine triacetate [41] and the 1050-Da glycosylated MAA from *N. commune* [29] (Table 8), which suggested that the 612-Da MAA had a similar structure to the known MAAs. The characteristic absorption peak at 3370 cm^−1^, but not that at 3310 cm^−1^, in the 612-Da MAA suggested the presence of a large number of hydroxyl groups, and the absorption peaks in the range of 1200–1300 cm^−1^ suggested the presence of sugars, which was consistent with the presence of two hexose rings as described below. The absorption peak at 1542 cm^−1^ suggested the presence of the conjugated imine in the 612-Da MAA.

**Figure 5:**
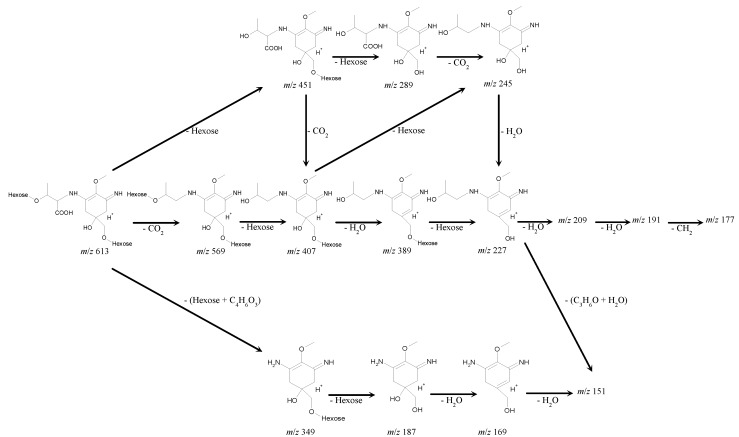
A proposed fragmentation pattern for the 612-Da MAA, based on the MALDI-TOF MS/MS analysis. MALDI-TOF MS/MS analysis was performed on the parent molecular ion fragment with *m*/*z* 613 and the other main molecular ion peaks with *m*/*z* 569, 451, 407, 389 and 349. With regard to the mass spectra, a plausible fragmentation pattern shows the presence of a two-hexose and a threonine chain linked by a cyclohexenimine ring.

MALDI-TOF MS/MS analysis was performed on the parent molecular ion fragment with *m*/*z* 613 and on the other main molecular ion fragments with *m*/*z* 569, 451, 407, 389 and 349 (Table 7). A plausible fragmentation pattern could be suggested with regard to the mass spectra (Figure 5). The ion fragment with *m*/*z* 569 indicated the deletion of CO_2_ from the parent molecular ion fragment with *m*/*z* 613, suggesting the presence of a carboxyl group. Subsequent deletions of 162 and 18 Da from the fragment with *m*/*z* 569 to produce the fragments with *m*/*z* 407 and *m*/*z* 389 indicated the deletions of hexose (C_6_H_10_O_5_) and H_2_O, respectively. MS/MS analysis of the fragments with *m*/*z* 407 and 389 led to the fragment at *m*/*z* 227, which indicated the deletion of another hexose (Table 7 and Figure 5). 

**Table 8:** Comparison of IR spectra of the 612-Da MAA with known MAAs.

612-Da MAA with A_max_ at 322 nm	Palythine triacetate	1050-Da MAA with A_max_ at 312 and 340 nm
Wavenumber (cm^−1^)		
3370		3399
	3310	
2932		2929
	1725, 1740	
	1660	
1607	1590	1617
1542	1535–1555	1541
1388		1400
1346		
1277, 1216		1275
1150		
1067, 1036		1076, 1046
972		

IR spectra were recorded with a Fourier transform infrared (FT-IR) spectrometer (Nicolet NEXUS 470 FT-IR) according to the KBr disk method. The wavenumbers of absorption bands for the 612-Da MAA are compared with those for the known MAA palythine triacetate [41] and the 1050-Da MAA [29]. The IR spectrum of the 612-Da MAA is shown in Figure 3b.

The successive deletions of 162 Da from the parental molecular fragment with *m*/*z* 613 to produce *m*/*z* 451 and *m*/*z* 289 indicates the presence of two hexoses (C_6_H_10_O_5_) in the parent molecular ion with *m*/*z* 613; the resultant fragment with *m*/*z* 289 can be assumed to be a palythine-threonine (C_12_H_20_N_2_O_6_ + H^+^). Consistent with this assumption, the deletion of 44 Da from *m*/*z* 289 to produce *m*/*z* 245 indicated the deletion of CO_2_ and suggested the presence of a carboxyl group in the molecular fragment with *m*/*z* 289. The fragment with *m*/*z* 227 could lose two molecules of H_2_O and CH_3_ to produce the fragments with *m*/*z* 209, 191 and 177, respectively. The fragment with *m*/*z* 227 might also lose a part of the threonine chain (C_3_H_6_O) and H_2_O to produce the fragment with *m*/*z* 170 (or *m*/*z* 169) and 151 (Table 7 and Figure 5). 

In a MS/MS analysis of the molecular ion fragment with *m*/*z* 349, the neutral loss of 162 Da indicated the presence of hexose in the fragment with *m*/*z* 349, and the fragments with *m*/*z* 169 and *m*/*z* 151 suggested the subsequent deletions of two H_2_O molecules (Table 7 and Figure 5). 

These fragmentation patterns were similar to the results from MS analyses of the known MAAs [42,43] and confirmed the proposed structure of the 612-Da MAA with the presence of two hexoses and a carboxyl group. 

**Figure 6:**
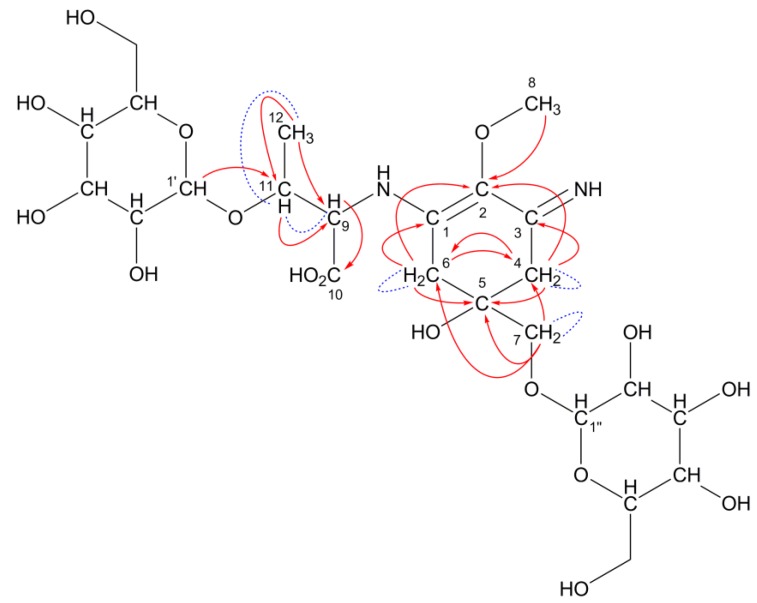
A predicted structure for the 612-Da MAA with an absorption maximum at 322 nm and a molecular formula of C_24_H_40_N_2_O_16_. Two hexose sugars are bound to palythine-threonine at C7 and C11 positions. Red arrows and blue dashed lines represent the apparent HMBC and COSY correlations, respectively.

^1^H, ^13^C and 2D NMR experiments were performed in D_2_O. The ^13^C and ^1^H NMR spectra of the 612-Da MAA were compared to those of the known MAAs mycosporine-glycine and palythine-threonine sulfate as well as β-d-glucose and methyl β-d-glucose (Table 9). Signals with identical chemical shifts to 3-aminocyclohexenimine (C1, 2, 3, 4, 5, 6) and threonine (C9, 10, 11, 12) were observed in the 612-Da MAA, similar to those of palythine-threonine sulfate. In mycosporine-glycine the chemical shifts assignable to carbonyl and imine carbon are obviously distinguishable (159.7 ppm and 187.2 ppm in Table 9, [44]). However, the chemical shifts corresponding to imine carbon (C3) and amino alkene carbon (C1) in the 612-Da MAA overlapped each other because of the conjugation between them. In palythine-threonine sulfate these chemical shifts assigned to C1 and C3 carbons also overlapped each other (Table 9, [45]). Therefore, high-resolution ^13^C NMR measurement focusing on the X-range at around 160 ppm was performed to separate two distinct signals assignable to imine and amino alkene carbons at C1 and C3 positions in the 612-Da MAA (NMR data shown in Supporting Information). These two separated signals demonstrated the presence of the 3-aminocyclohexenimine ring in the 612-Da MAA. In the 2D NMR spectra, characteristic correlations assignable to the MAA backbone were detected. The correlations in COSY and HMBC spectra are summarized in Table 10 and shown in Figure 6 as red arrows and blue dashed lines.

**Table 9:** Summary of the NMR analysis of the 612-Da MAA with an absorption maximum at 322 nm.

C	612-Da MAA with A_max_ at 322 nm		Palythine-threonine sulfate		Mycosporine-glycine	
	^13^C	^1^H	^13^C	^1^H	^13^C	^1^H
1^ a^	163.6	-	160.6	-	159.7	-
2	127.7	-	125.0	-	130.4	-
3^ a′^	163.6	-	160.6	-	187.2	-
4^ b^	37.0	2.85, 3.10 (ABq, 17.6)	34.0	2.81 (ABq, 17.0)	45.4	2.50, 2.73 (ABq, 17.0)
5	73.3	-	69.8	-	72.9	
6^ b′^	38.9	2.76, 3.03 (ABq, 17.0)	36.0	2.77 (ABq, 17.0)	33.8	2.72, 2.83 (ABq, 17.0)
7	76.0	3.49 (d, 10.0), 3.78 (d, 10.0)	72.5	3.90 (s)	68.4	3.57 (s)
8	62.1	3.69 (s)	59.3	3.55 (s)	60.2	3.64 (s)
9	66.4	4.17 (d, 4.6)	64.5	3.98 (d, 4.5)	43.7	4.24 (s)
10	177.3	-	174.9	-	174.5	-
11	75.7	4.34 (dq, 4.6, 6.4)	68.2	4.20 (dq, 4.5, 6.0)		
12	18.9	1.28 (d, 6.4)	19.5	1.12 (d, 6.0)		
			Methyl β-d-Glc		β-d-Glc	
			^13^C	^1^H	^13^C	^1^H
1′	107.4	5.09 (s)	104.0	4.27	96.8	4.64
1″	101.7	5.00 (d, 3.7)				

NMR spectra were recorded with a JEOL ECS400 spectrometer in D_2_O as a solvent. Chemical shifts (ppm) for the purified 612-Da MAA with A_max_ at 322 nm are compared with those for the known MAA mycosporine-glycine [44], palythine-threonine sulfate [45] and methyl β-d-Glc and β-d-Glc [39]. The coupling patterns and constants (Hz) are shown in parentheses. x, x′, Chemical shifts may be exchangeable for palythine-threonine sulfate and the 612-Da MAA.

**Table 10:** Characteristic correlations in COSY and HMBC spectra assignable to the 612-Da MAA backbone.

	COSY	HMBC
H4	H4	C2, C3, C5, C6
H6	H6	C1, C2, C4, C5
H7	H7	C4, C5, C6
H8		C2
H9	H11	C10
H11	H9, H12	C9
H12	H11	C9, C11
H1′		C11

Two separable signals that are characteristic for the anomer positions (C1′ and C1″) of distinguishable carbohydrates were observed (Table 9), demonstrating the presence of two carbohydrates. The downfield ^13^C chemical shifts of the carbohydrate anomer positions suggested hexose sugars bound to the palythine-threonine scaffold of the 612-Da MAA. As described in the section on the structure determination of the 508-Da MAA, the downfield ^13^C shifts of C7 and C11 were thought to be due to hexose binding via *O*-glycoside bonds in the 612-Da MAA. According to the HMBC spectrum, a correlation between the C11 carbon in the MAA backbone and the proton bound to the anomer carbon (C1′) of hexose was observed, suggesting the presence of hexose bound at the C11 position. The correlation between the C7 carbon and the anomer proton of the other hexose did not appear; however, geminal coupling at the C7 methylene protons due to the high rotational barrier was observed, suggesting the presence of a large functional group such as a hexose at the C7 position. The difference of ^13^C chemical shift at C7 position between the 612-Da MAA and palythine-threonine sulfate was only 3.5 ppm, which is smaller than that expected from the difference between the 508-Da MAA and porphyra-334. Because a sulfate group is a strong electron-withdrawing substituent, the ^1^H-chemical shift at the C7 position in palythine-threonine sulfate shifts downfield from that in palythine-threonine. 

These results are consistent with the MS analysis of the 612-Da MAA. After combining the data from the MS and NMR analysis, the predicted structure of the 612-Da MAA was generated and is shown in Figure 6. 

### 2.4. 450-Da MAA with an Absorption Maximum at 322 nm

Another MAA with an absorption maximum at 322 nm and a different retention time in the HPLC analysis elution profile (Figure 1) was purified from the same *N. commune* colonies (Table 1, Figure S1). The UV absorption spectrum of the purified MAA showed a single absorption peak at 322 nm (Figure S1). A molecular mass of 450 Da was determined by MALDI-TOF MS and FAB MS. MALDI-TOF MS/MS analysis was performed on the parent molecular ion fragment with *m*/*z* 451 and the other main molecular ion fragments with *m*/*z* 407, 389 and 349. The fragmentation patterns were similar to those of the 612-Da MAA and suggested the presence of a hexose and the same palythine-threonine scaffold (Table 11). The ^1^H and ^13^C NMR analyses suggested that the structure consisted of a cyclohexenimine chromophore that was similar to that of the 612-Da MAA with one hexose sugar (data not shown). These data suggest that the 450-Da MAA is a hexose-bound palythine-threonine. Additionally, MALDI-TOF MS analysis suggested that the hexose was not bound to the threonine chain (Table 11). 

**Table 11:** Summary of MALDI-TOF MS analysis of the purified 450-Da MAA with absorption maximum at 322 nm.

	Mass of fragment	Relative abundance	Neutral loss	Deleted fragment
MS of purified MAA	451.22	82		
	407.23	100	44	CO_2_
	389.22	53	62	CO_2_ + H_2_O
	349.18	29	102	C_4_H_6_O_3_
MS^2^ of *m*/*z* 451	**451.22**			
	289.21	100	162	Hexose
	245.18	13	206	Hexose + CO_2_
	185.16	7	266	-
	91.09	8	360	-
MS^2^ of *m*/*z* 407	**407.23**			
	245.23	100	162	Hexose
	230.20	7	177	Hexose + CH_3_
	227.21	8	180	Hexose + H_2_O
	213.21	15	194	Hexose + H_2_O + CH_2_
	211.23	12	196	-
	209.18	7	198	Hexose + 2H_2_O
	199.18	16	208	Hexose + C_2_H_6_O
	197.21	16	210	-
	185.17	20	222	-
	177.17	11	230	Hexose + 3H_2_O + CH_2_
	169.17	5	238	Hexose + H_2_O + C_3_H_6_O
	151.16	7	256	Hexose + 2H_2_O + C_3_H_6_O
	91.09	8	316	-
MS^2^ of *m*/*z* 389	**389.22**			
	227.19	100	162	Hexose
	209.18	16	180	Hexose + H_2_O
	191.16	13	198	Hexose + 2H_2_O
	181.16	14	208	Hexose + C_2_H_6_O
MS^2^ of *m*/*z* 349	**349.09**			
	187.18	100	162	Hexose
	169.16	8	180	Hexose + H_2_O
	151.15	18	198	Hexose + 2H_2_O

### 2.5. Radical Scavenging Activity in Glycosylated MAAs

Table 12 shows the radical scavenging activity found in the purified 508-Da and 612-Da MAAs from *N. commune*. Both the 508-Da MAA and the 612-Da MAA showed ABTS radical scavenging activity as determined by the decolorization of ABTS radicals. During the time course experiments, the decolorization of the ABTS radicals increased as the incubation time extended from 10 min to 2 h, which suggested that these MAAs were slow-acting radical scavengers. Trolox and ascorbic acid, which were used as standards, are known to be fast-acting scavengers that bring the decolorization reactions to completion within 10 min. Based on these results, the assay incubation time was fixed at 1 h as described by Matsui *et al.* [29]. When ESR was used to directly monitor the decrease in ABTS radical concentrations, the activity of the 612-Da MAA was comparable to that of Trolox and ascorbic acid, while the 508-Da MAA showed weak activity (Table 12). 

**Table 12:** Radical scavenging activity in MAAs ^a^.

Assay	Colorimetry ^b^	ESR ^c^
	IC_50_ (mM)	
Ascorbic acid ^d^	0.28	0.16
Trolox ^d^	0.25	0.16
508-Da MAA with A_max_ at 334 nm	58	29
612-Da MAA with A_max_ at 322 nm	16	0.25

^a^ Radical scavenging activity was measured with ABTS as the organic radical source; ^b^ Decolorization of ABTS was monitored with a spectrophotometer for 1 h; ^c^ ESR signals were monitored with a free radical monitor (JEOL JES-FR30EX); ^d^ Ascorbic acid and Trolox were used as standards; IC_50_ (50% inhibitory concentration) values are shown.

**Figure 7:**
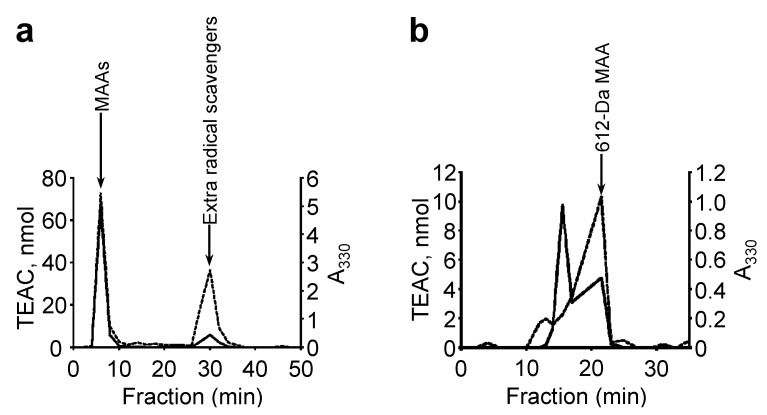
The elution profile of a water extract from *N. commune* that was fractionated by HPLC. MAAs were detected at A_330_ (solid line). The radical scavenging activity (TEAC) was measured by the ABTS decolorization assay (dashed line). (**a**) The water extract was separated on a reverse phase column as described in the Experimental Section. The MAAs were eluted together with their associated radical scavenging activities in the fraction at 6 min, and additional radical scavenging activity was detected in the fraction at 30 min; (**b**) The MAA-containing fraction collected at 6 min was additionally separated on a gel filtration column as described in Experimental Section. The 612-Da MAA was eluted together with its associated radical scavenging activity from 17 to 21.5 min in a volume of 2.25 mL.

The MAA-associated radical scavenging activity was examined in the *N. commune* water extract by HPLC fractionation (Figure 7a). The fraction with MAA-associated radical scavenging activity accounted for approximately 45% of the total recovered activity (Figure 7a). Interestingly, high radical scavenging activity, which was not MAA-associated, was detected and determined to account for approximately 40% of the total recovered activity (Figure 7a). This result suggested that additional water-soluble antioxidants contributed highly to the total radical scavenging capacity of genotype D *N. commune*. This non-MAA radical scavenger will be characterized in future studies. 

The recovered fractions with MAAs were further examined on an HPLC system with a gel filtration column (Figure 7b). The 612-Da MAA accounted for approximately 60% of the total MAAs, and the radical scavenging activity eluted with the 612-Da MAA accounted for approximately 45% of the recovered activity (Figure 7b). These results suggest that the 612-Da MAA is a major MAA in genotype D *N. commune* and is also a main component of the water-soluble radical scavengers, as it contributed approximately 20% of the total water-soluble radical scavenging activity in the water extract. 

### 2.6. Specificity of the Genotypes and MAAs

*N. commune* is known to be genetically diverse, and four major genotypes of *N. commune* have been reported in Japan; however, the morphological features of the macroscopic colonies and microscopic trichomes are almost identical, and the genotypes are indistinguishable without a determination of molecular taxonomical markers [33]. During an investigation of MAA contents in field-isolated *N. commune* colonies, we observed different UV-absorption spectra in the water extracts from colonies at different sampling locations. We have reported two types of *N. commune*, those that specifically produce a 478-Da MAA with an absorption maximum at 335 nm or those that produce a 1050-Da MAA with double absorption maxima at 312 and 340 nm, although biological or physiological differences that would allow us to separate the different MAA producers could not be determined [29]. In addition to these two types of *N. commune* colonies, another type of *N. commune* colony, the water extract of which showed a characteristic UV-absorbing spectrum with an absorption maximum at 325 nm was found. According to the unique UV-absorbing spectrum and HPLC chromatogram of the water extract, we thought that this particular *N. commune* contained a structurally different MAA. Thus, in this study, the novel glycosylated MAAs were purified and characterized from organisms that did not produce either the 478-Da MAA or the 1050-Da MAA. Eight samples of *N. commune* from different sampling locations that had an absorption maximum at 325 nm in their water extracts were identified as genotype D, according to their 16S rRNA nucleotide sequences [46]; these data confirmed that genotype D is the 612-Da MAA producer*.* The glycosylated MAA patterns could be a feasible chemotaxonomic marker with which to characterize *N. commune*; it might be classified into three groups, namely, the 478-Da MAA producer, the 1050-Da MAA producer and the 612-Da MAA producer, as these groups are genetically different chemical races of *N. commune*. Further investigations into the genotypes and specificities of the types of the glycosylated MAAs are ongoing, and the results will be published in the future. 

### 2.7. Glycosylated MAAs

The chemical structures were determined for two glycosylated MAAs that were previously identified in *N. commune* [29]. The first was the 478-Da MAA with an absorption maximum at 335 nm that was identified as a pentose-bound porphyra-334 derivative. The second MAA, with a molecular mass of 1050 Da, had a characteristic UV absorption spectrum with an absorption peak at 312 nm, which was associated with a shoulder at 340 nm. The 1050-Da MAA is unique due to its high molecular mass and the presence of both 3-aminocyclohexen-1-one and 3-aminocyclohexenimine chromophores within a single molecule. These unique structural features with different chromophores are related to the expanded UV-absorbing window due to the double absorption maxima that cover both the UV-A and UV-B wavelengths. Interestingly, all of the MAAs characterized so far in *N. commune* have been glycosylated [29,47]. This glycosylation is unique to the terrestrial cyanobacterium *N. commune*, although the function of this glycosylation has not yet been clarified. However, other glycosylated mycosporines, including mycosporine-glutaminol-glucoside and mycosporine-glutamicol-glucoside, have been reported in rock-inhabiting microcolonial fungi [48] and terrestrial cyanobacteria from rock surfaces [49]. The high concentrations of mycosporine-glutaminol-glucoside in desert rock-inhabiting fungi might be related to their survival potential in a terrestrial environment [50]. Hence, the glycosylated MAAs might be generally protective and allow adaptation to terrestrial environments in which the organisms are exposed to drastic changes in temperature and extreme desiccation, as well as direct solar radiation in the biologically harmful UV range. The details of these ecophysiological roles of the glycosylated MAAs remain to be demonstrated directly in future studies. 

### 2.8. Porphyra-334 and Its Derivatives

Porphyra-334, a UV-protective compound with absorption maximum at 334 nm and molecular mass of 346 Da, was first identified in the marine red alga *Porphyra tenera* [51] and was reported to be one of the most common MAAs in marine algal species [52]. To date, a limited number of studies have been published on the occurrence of porphyra-334 in cyanobacteria. Porphyra-334 has been reported in the marine cyanobacterial species of *Nodularia* [53], in the freshwater bloom-forming cyanobacterium *Microcystis aeruginosa* [54], in the aquatic cyanobacterium *Aphanizomenon flos-aquae* [35] and in the rice-field cyanobacterium *Anabaena doliolum* [55]. We have reported a 478-Da MAA pentose-bound porphyra-334 derivative in the terrestrial cyanobacterium *N. commune* [29], and in this study, we have identified a 508-Da MAA to be a hexose-bound porphyra-334 derivative. These studies have indicated the existence of glycosylated porphyra-334 in *N. commune*; however, the non-glycosylated form of porphyra-334 was not observed and the glycosylation of porphyra-334 in *N. commune* suggests a unique adaptation for terrestrial environments.

**Figure 8:**
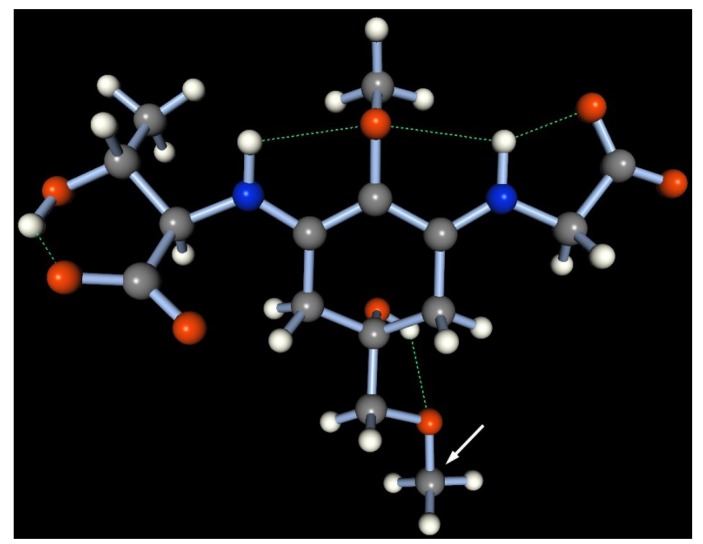
A stable geometry of methyl porphyra-334 obtained by a molecular simulation of MOPAC 2011, based on the PM6 Hamiltonian function. The hydrogen bond is represented as a green dashed line. Gray: carbon, white: hydrogen, blue: nitrogen, red: oxygen atom. A methyl group (used instead of a hexose group) is indicated by a white arrow.

The absorption maximum of the hexose-bound porphyra-334 derivative from *N. commune* reversibly shifted to 332 nm in a highly acidic solution, similar to porphyra-334 [56], which suggested that glycosylated porphyra-334 is a zwitterion in aqueous solution and thus is stable across a wide pH range. To confirm the stable conformation, a semi-empirical molecular simulation of the 508-Da MAA in neutral water at 25 °C was performed by molecular orbital package (MOPAC) 2011 with the PM6 Hamiltonian function (Figure 8). A methyl group was substituted to mimic the hexose group in the 508-Da MAA and simplify the simulation (indicated by a white arrow in Figure 8). The most stable form of the methyl-porphyra-334 derivative occupied a near-planar conformation, which allowed for a stable conformation with three hydrogen bonds in the molecule (depicted as green dashed bonds; heat of formation = −485.75 kcal/mol). In acidic conditions, the protonation on the carboxylate anion could cleave the one of the hydrogen bonds and thus would slightly prevent the resonance delocalization of π-electrons within the molecule. 

The biosynthesis of porphyra-334 is thought to be genetically controlled, and porphyra-334 producers can be classified as a taxonomical group in cyanobacteria and also in marine algae. Biochemical and molecular biological studies of the biosynthesis of the porphyra-334 scaffold based 478-Da and 508-Da MAAs in *N. commune* will yield further understanding of the diversity and function of porphyra-334, which is produced and accumulated in taxonomically diverse marine, freshwater and terrestrial organisms [18,19,20]. 

### 2.9. Palythine-Threonine and Its Drivatives

Palythine-threonine, an MAA with absorption maximum at 320 nm and molecular mass of 288 Da, was first identified in the corals *Pocillopora capitata*, *Stylophora pistillata* and *Pocillopora eydouxi* [42], however its sulfate ester had been reported in the reef-building coral *S. pistillata* [45]. To date, no studies have been published on the occurrence of palythine-threonine in cyanobacteria. In this study, we have identified the 612-Da MAA consisting of a cyclohexenimine chromophore conjugated with the substituent group of threonine (Figure 6), and this scaffold was characterized as a palythine-threonine with a molecular mass of 288 Da. This is the first report of the unique glycosylated palythine-threonine, which was not listed for previously reported MAAs [18,19,22,57,58]. The absorption maximum of this molecule at 322 nm (Figure 2b), the MS/MS results (Table 7) and the molecular mass of its scaffold were similar to those of the previously reported MAA palythine-threonine [42]. The identification of the glycosylated palythine-threonine from *N. commune* provides new insight into the molecular diversity of MAAs and the glycosylation of palythine-threonine in the terrestrial cyanobacterium suggests a unique adaptation for terrestrial environments that are harsh and drastically fluctuating in comparison to stable aquatic environments. However, the biosynthesis and glycosylation of palythine-threonine in *N. commune* remain to be elucidated in future studies. Biochemical and molecular biological studies of the biosynthesis of palythine-threonine in cyanobacteria will yield further understanding of this unique MAA originally identified in corals. 

### 2.10. Antioxidative Role of MAAs

In terrestrial environments, *N. commune* colonies are subjected to desiccation and UV-irradiation. Protective compounds that are involved in reactive oxygen-scavenging mechanisms must have an important role in increasing cell tolerance to the oxidative stresses associated with desiccation and UV irradiation [12,59]. MAAs have been suggested to be protective against UV-induced oxidative stress in algae [60] and in cyanobacteria [61]. In *N. commune*, the 478-Da and 1050-Da MAAs were reported to show radical scavenging activity *in vitro*, and the 1050-Da MAA, which has potent radical scavenging activity, contributes approximately 27% of the total water-soluble radical scavenging activity [29]. The glycosylated MAAs identified in this study also showed radical scavenging activities *in vitro* (Table 12). Although porphyra-334 was reported to have photoprotective but not antioxidant functions [62,63,64], both glycosylated derivatives of porphyra-334, the 508-Da MAA (Table 12) and the 478-Da MAA [29], had slow-acting radical scavenging activities *in vitro*. The glycosylation of porphyra-334 has been suggested to provide the antioxidant activities of these glycosylated porphyra-334 derivatives, although the molecular mechanism is unknown. The 612-Da MAA was the main water-soluble radical scavenger in the water extract, as it provided approximately 20% of the water-soluble radical scavenging activities in *N. commune*. The 612-Da and 1050-Da MAAs were the major MAAs in their respective colonies and demonstrated very similar water-soluble radical scavenging capacities [29]. The high *in vitro* radical scavenging activity of the 1050-Da MAA consisting of cyclohexenone chromophores is consistent with the antioxidative functions of the oxocarbonyl-type MAAs, such as mycosporine-glycine [64,65,66] and mycosporine-taurine [61]. Because the 612-Da MAA is not an oxocarbonyl-type MAA, the high radical scavenging activity may be due to its glycosylation, similar to the glycosylated porphyra-334 derivatives. These findings suggest the importance of these glycosylated MAAs to the oxidative stress response in *N. commune* and support the idea of multifunctional MAAs as UV-protectants and antioxidants. 

### 2.11. Localization of Glycosylated MAAs in *N. commune*

The 36-kDa water stress protein (WspA) accounts for more than 70% of the extracellular matrix protein content of *N. commune* [16,17,67]. WspA binds to the scytonemin and MAA through non-covalent interactions and is assumed to be relevant to the structure and/or the function of the extracellular matrix [15,16,68]. UV irradiation stimulates the synthesis and secretion of WspA [16], as well as EPS production and scytonemin and MAA synthesis [69]. The xylose-containing MAA, which interacts with EPS via its sugar moieties, was reported to form multimeric complexes with WspA through strong ionic interactions in the absence of salt, which must be subject to the attenuation of UV-induced radiation damage during desiccation in *N. commune* [16,68]. MAA glycosylation might be relevant to the architecture of the extracellular matrix in *N. commune*, but the details must be characterized in future studies. 

### 2.12. Biosynthesis of Glycosylated MAAs in *N. commune*

**Figure 9:**
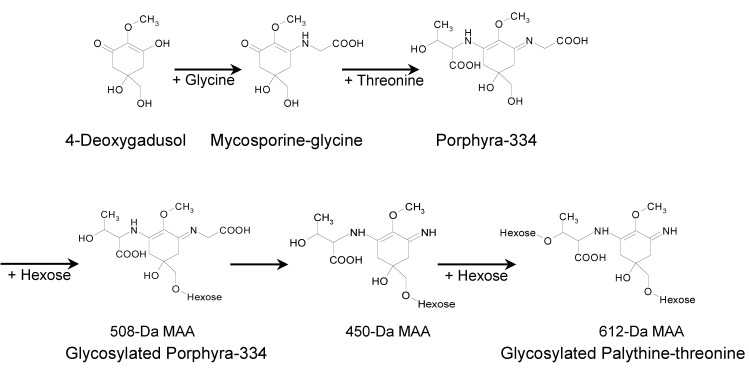
A plausible biosynthetic pathway for glycosylated MAAs in *N. commune* (genotype D).

More than 30 structurally distinct MAAs, including the mycosporine-derivatives, have been characterized to date [18,19,22,57,58]. These MAAs are formed from the precursor 4-deoxygadusol [70] and are conjugated with a nitrogen substituent (e.g., amino acids) [18,19,22,71]. The gene product of Ava_3856 from *Anabaena variabilis* can convert 4-deoxygadusol and glycine into mycosporine-glycine [70], which is a metabolic precursor of the bi-substituted MAAs [70,72]. Previous reports indicate that condensation of the mono-substituted mycosporine-glycine with an amino acid would be a common reaction in the generation of bi-substituted MAAs such as shinorine and porphyra-334 [70,72]. Consistent with a previous report on the coral *P. capitata* which suggested that palythine-threonine is formed by decarboxylation of porphyra-334 followed by demethylation of mycosporine-methylamine-threonine [42], the simultaneous occurrence of palythine-threonine and porphyra-334 as scaffolds in *N. commune* suggests that porphyra-334 could be a metabolic precursor of palythine-threonine (Figure 9). Supporting this idea, the glycosylated palythine-threonine accounted for 60% of the total MAAs and was the most abundant one in *N. commune*. According to the structures and fragmentation patterns of the glycosylated MAAs, a MAA biosynthetic pathway is predicted in *N. commune* (Figure 9). In our predicted pathway, porphyra-334 could be generated via the addition of threonine to the core ring of mycosporine-glycine and the glycosylation of porphyra-334 could produce the 508-Da MAA. The 450-Da MAA could be formed via the elimination of a portion of glycine from C3 of the 508-Da MAA. The addition of another hexose to the 450-Da MAA could produce the 612-Da MAA (Figure 9). This biosynthetic pathway for the production of the glycosylated palythine-threonine and porphyra-334 derivatives could occur particularly in genotype D of *N. commune*; however, the enzymes and genes involved in the biosynthesis of these glycosylated MAAs are unknown. Further molecular genetic studies are required to identify the enzymes and associated genes in the predicted biosynthetic pathway as well as the function of these glycosylated MAAs in adaptation to terrestrial environments.

## 3. Experimental Section

### 3.1. Microorganisms

Colonies of field-grown *N. commune* were collected from the Kakuma Campus of Kanazawa University (N 36.544812, E 136.709635), Ishikawa, Japan. Wet colonies that were naturally swollen in response to rain were harvested, washed with tap water to remove soil, air-dried in the laboratory, and stored at room temperature until used. Water extracts from *N. commune* powders were examined spectrophotometrically to confirm the absorption maximum at 325 nm, and genotypes were characterized by the nucleotide sequence of the 16S rRNA gene as described previously [33].

### 3.2. Purification of MAAs

*N. commune* powder (30 g) that contained MAAs with absorption maxima at 334 or 322 nm was suspended in distilled water (2 L), and the MAAs were extracted by stirring at room temperature for 2 h. After centrifugation at 15,240× *g* for 20 min at 4 °C, the supernatant was vacuum-filtered with a Buchner sintered-glass filter funnel and then condensed to approximately 600 mL with a rotary evaporator under reduced pressure. Sufficient ethanol was added to the filtrate to yield a final concentration of 70% ethanol (v/v), and the mixture was kept at 4 °C for 1 h in the dark to precipitate the 70% ethanol-insoluble materials. After centrifugation at 15,240× *g* for 20 min at 4 °C, the supernatant was vacuum filtered with a Buchner sintered-glass filter funnel. The filtrate was evaporated and centrifuged at 21,500× *g* for 10 min at 4 °C. The supernatant was filtered through a 0.20-μm syringe filter (Minisart RC 15, Sartorius Stedim, Göettingen, Germany) and injected into an HPLC system with a Hitachi L-6200 pump that was equipped with a reverse phase column (IRICA C18, 20 × 250 mm). The mobile phase changed stepwise from distilled water during the first 40 min to 10% (v/v) methanol with 0.1% (v/v) acetic acid during the next 20 min to 100% methanol during the final 20 min. The flow rate was kept at 4 mL·min^−1^, and the A_330_ was monitored with a Hitachi L-4200 UV-VIS detector. The fractions with the MAAs were recovered separately, condensed with a lyophilizer and injected into an HPLC system equipped with a gel filtration column (TSKgel G2500PW, TOSOH, Tokyo, Japan). The mobile phase was water at a flow rate of 1 mL·min^−1^, and A_330_ was monitored with a Hitachi L-4200 UV-VIS detector. The MAA fractions were recovered, and the final MAA products were lyophilized. In each step, the total MAA concentration was estimated spectrophotometrically with an extinction coefficient of 120 L·g^−1^·cm^−1^ [34]. To determine the extinction coefficients of the purified MAAs with absorption maxima at 334 or 322 nm, diluted solutions were prepared in water, and the A_334_ or A_322_ was determined, respectively. The MAA dry weight in 1 mL of solution was measured after lyophilization. 

### 3.3. MS Analysis

MALDI-TOF MS analysis was performed at the Division of Functional Genomics, Advanced Science Research Center, Kanazawa University, on a tandem mass spectrometer (4800 plus MALDI TOF/TOF™ Analyzer; Applied Biosystems, Foster City, CA, USA) with 2,5-dihydroxybenzoic acid (DHB) as a matrix. The secondary mass spectrum was recorded when applicable. FAB MS analysis to determine the accurate mass and predicted elemental composition was performed at the Research Institute for Instrumental Analysis in Kanazawa University on a mass spectrometer (JMS-SX102A, JEOL, Tokyo, Japan) with glycerol as a matrix.

### 3.4. Spectroscopic Methods

UV-VIS spectra were recorded with a Hitachi U-2800 spectrophotometer. Fourier transformation infrared (FT-IR) spectra were recorded with a Nicolet NEXUS 470 FT-IR by the KBr disk method. NMR spectra in a D_2_O solvent were recorded with a JEOL ECS400 spectrometer at the Research Institute for Instrumental Analysis in Kanazawa University. 3-(Trimethylsilyl)-1-propanesulfonic acid-d6 sodium salt (TMP) was used as an internal NMR standard. In order to separate two overlapping signals assignable to imine carbons in the 612-Da MAA, high-resolution ^13^C NMR measurement was performed focusing on the X-range around 160 ppm and the NMR spectrum was recorded with a JEOL ECA-600 spectrometer.

### 3.5. Measurement of Trolox Equivalent Antioxidant Capacity (TEAC)

Radical scavenging activity was measured with 2,2′-azino-*bis*(3-ethylbenzothiazoline-6-sulfonic acid) (ABTS) as a substrate in a colorimetric assay [73]. Decolorization at A_734_ was monitored spectrophotometrically for 1 h. The electron spin resonance (ESR) signals of ABTS were recorded with a free radical monitor (JES-FR30EX, JEOL, Tokyo, Japan). Trolox (6-hydroxy-2,5,7,8-tetramethylchroman-2-carboxylic acid) and ascorbic acid were used as artificial and natural water-soluble antioxidant standards. 

### 3.6. Chromatographic Separation and Detection of MAAs and Radical Scavenging Activities in *N. commun*e Water Extracts

*N. commune* powder (1 g) was suspended in distilled water (100 mL) and extracted at room temperature by stirring for 4 h. After centrifugation at 21,500× *g* for 10 min at 4 °C, the supernatant was concentrated with a centrifugal concentrator (VC-360, TAITEC, Koshigaya, Japan) and filtered through a 0.20-μm syringe filter (Minisart RC 15, Sartorius Stedim). The concentrated water extract with 178 nmol TEAC was injected into an HPLC system with a Hitachi L-6200 pump and an L-4200 UV-VIS detector equipped with a reverse phase column (Wakosil 5C18, 4.6 mm × 250 mm; Wako, Osaka, Japan). The mobile phase changed stepwise from distilled water during the initial 14 min to 100% methanol during the next 36 min. The flow rate was kept at 0.5 mL·min^−1^, and a 1-mL fraction was collected every 2 min. The MAA-containing fraction with the highest radical scavenging activity was re-injected into another HPLC system with a Hitachi L-6200 pump and an L-4200 UV-VIS detector equipped with a TSKgel G2500PW column. The mobile phase was distilled water, and the flow rate was constant at 0.5 mL·min^−1^. The MAAs were detected at A_330_. Radical scavenging activity was measured by ABTS decolorization assay. 

## 4. Conclusions

In this study, novel glycosylated MAAs with radical scavenging activities were identified in the terrestrial cyanobacterium *Nostoc commune*. The chemical structures of these glycosylated MAAs were different from those of previously reported MAAs [18,19,22,29]. The major MAA, which had a molecular mass of 612 Da and an absorption maximum at 322 nm, was identified as a two-hexose-bound palythine-threonine derivative (Figure 6). The second MAA, which had a molecular mass of 508 Da and an absorption maximum at 334 nm, was identified as a hexose-bound porphyra-334 derivative (Figure 4). The MAA with a molecular mass of 450 Da and an absorption maximum at 322 nm was identified as a hexose-bound palythine-threonine derivative. The 450-Da and 612-Da MAAs were linked to one or two hexoses, and their scaffolds were related by a common palythine-threonine scaffold, suggesting that the 450-Da MAA is a likely intermediate from which the 612-Da MAA is produced via glycosylation (Figure 9), although there are no data to rule out the possibility of a break-down product of the 612-Da MAA. 

*N. commune* excretes and accumulates MAAs in the extracellular matrix, and thus far, all characterized MAAs in *N. commune* have been glycosylated. Putatively, *N. commune* would be incapable of surviving terrestrial environments and sustaining viability in a long-term desiccated state without glycosylated MAAs. The glycosylation mechanism and physiological roles of these glycosylated MAAs with antioxidative activities in this unique anhydrobiotic organism, as well as their interactions with other components of the extracellular matrix architecture, remain to be elucidated in future studies.

## Supplementary Files

Supplementary File 1 Supporting Information (PDF, 2994 KB)
