# Supplementary material for: Glycosylated Porphyra-334 and Palythine-Threonine from the Terrestrial Cyanobacterium Nostoc commune
Source: Mar Drugs. 2013 Aug 26;11(9):3124–54. doi: 10.3390/md11093124 (PMC3801118; doi:10.3390/md11093124)

Supporting Information

Figure S1. HPLC chromatograms and absorption spectra of the purified MAAs. (**a**) The crude water-soluble extract of *N. commune* (genotype D) was separated by an HPLC system equipped with a preparative column (IRICA C18, 20 × 250 mm) as shown in Figure 1. The mobile phase changed in a stepwise fashion from distilled water for the initial 40 min, to 0.1% acetic acid 10% methanol for the next 20 min and to 100% methanol for the final 20 min. The flow rate was constant at 4 mL min^{-1}, and the A_{330} was monitored. The purified 508-Da MAA (**b**), 450-Da MAA (**c**) and 612-Da MAA (**d**) were analyzed by HPLC with an analytical reverse-phase column (Inertsil ODS-3, 4.6 mm × 250 mm; GL Sciences Inc., Tokyo, Japan) using 0.2% acetic acid at a flow rate of 1 mL min^{-1} as the mobile phase and were detected by the A_{330}. The purified 508-Da MAA (**e**), 450-Da MAA (**f**) and 612-Da MAA (**g**) showed absorption maximum at 334 nm, 322 nm and 322 nm, respectively.

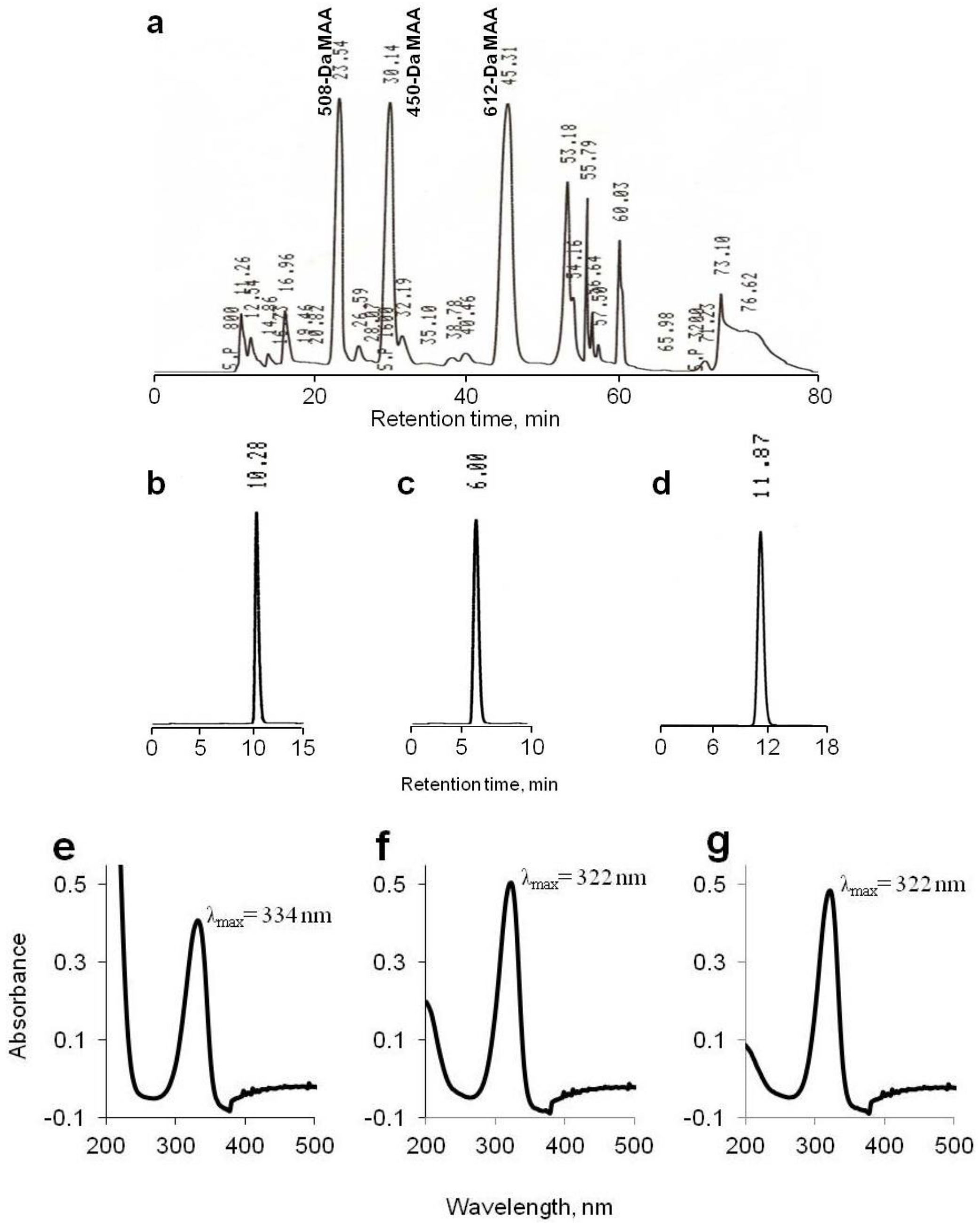

1. NMR Spectra for the Determination of Chemical Structures of the 508-Da MAA (Figures S2–S7) and the 612-Da MAA (Figures S8–S15)

1.1. NMR Data for the 508-Da MAA

Figure S2. ^{1}H NMR spectra of the 508-Da MAA in D_2O.

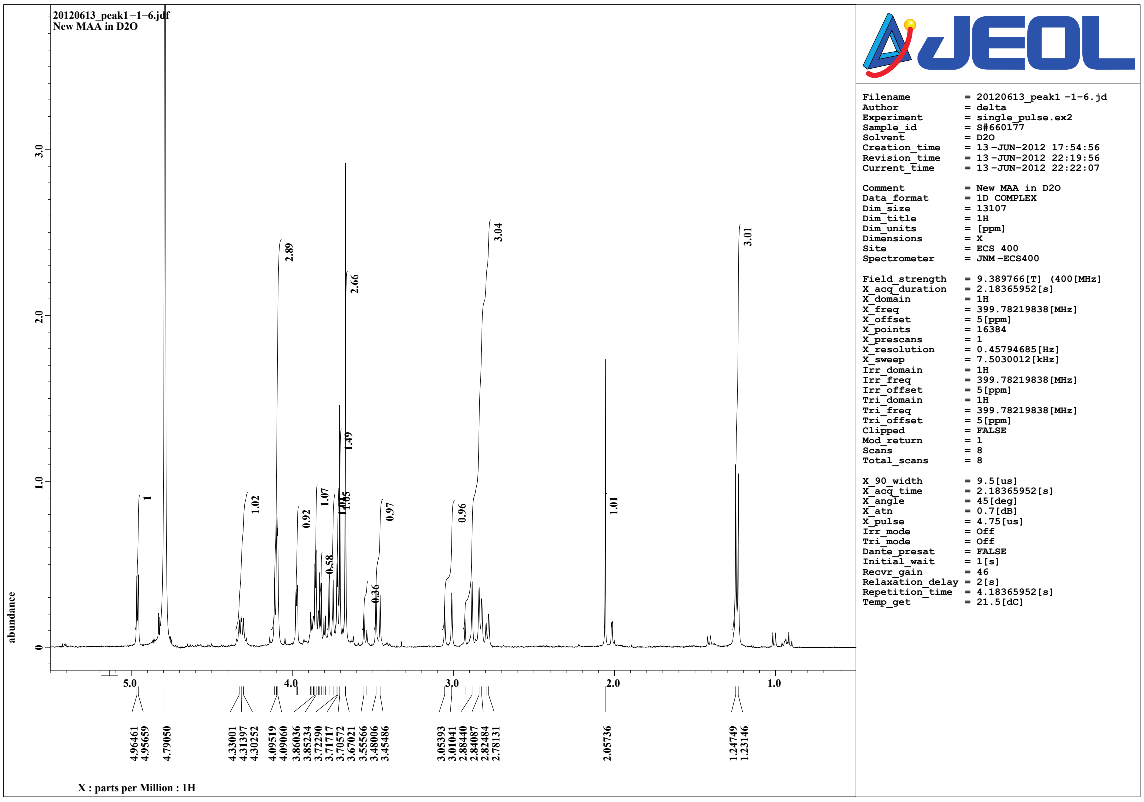

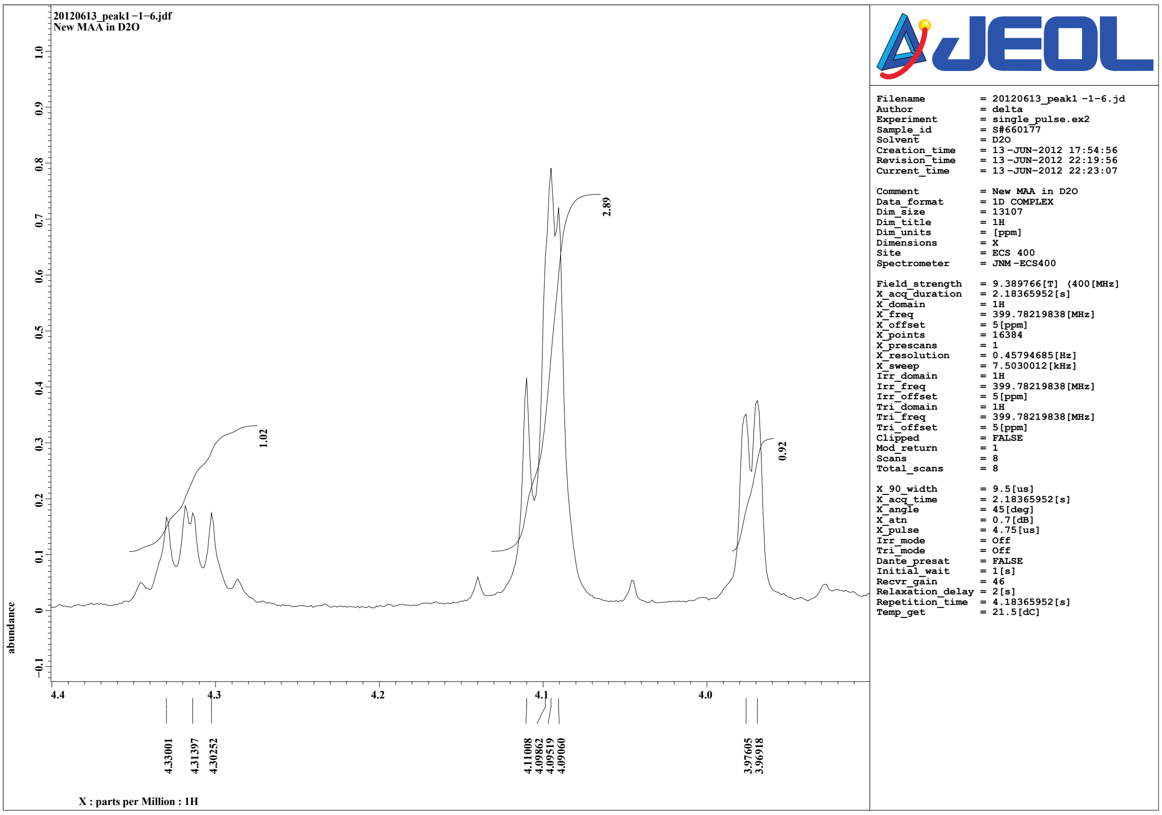

Figure S2. *Cont.*

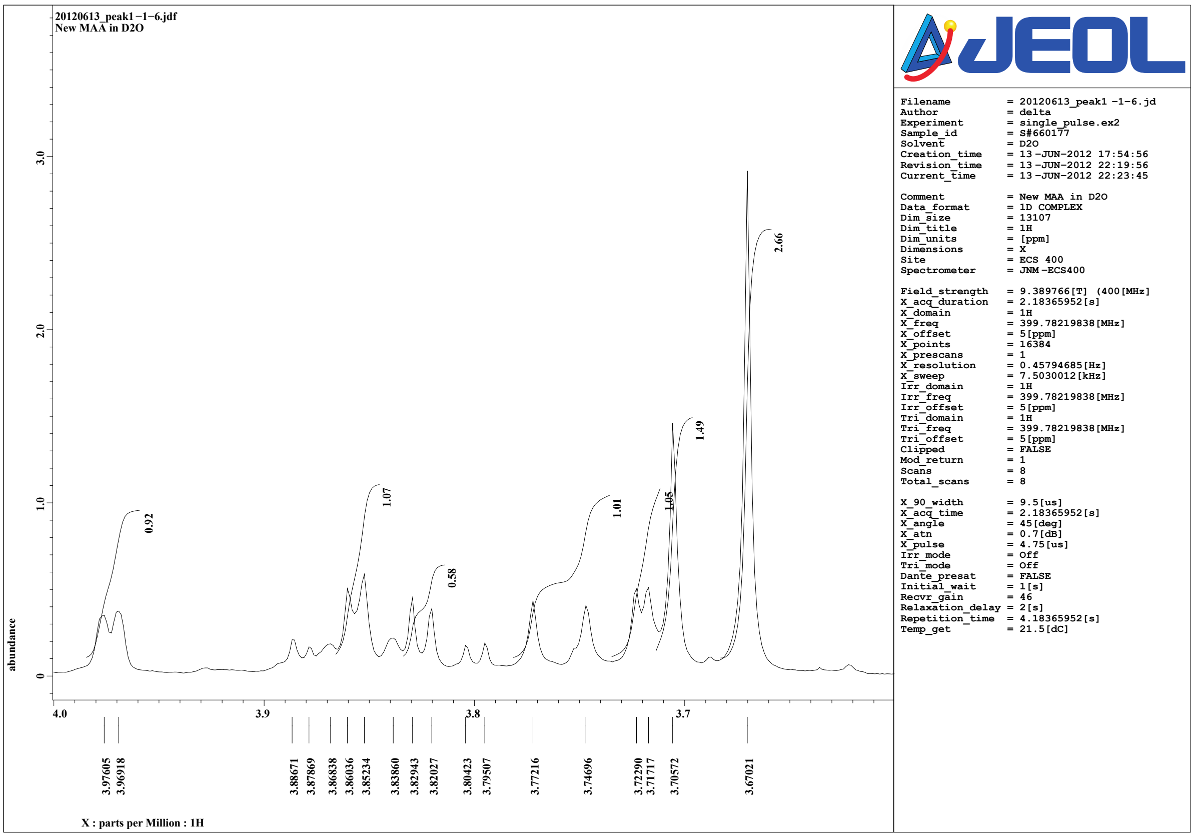

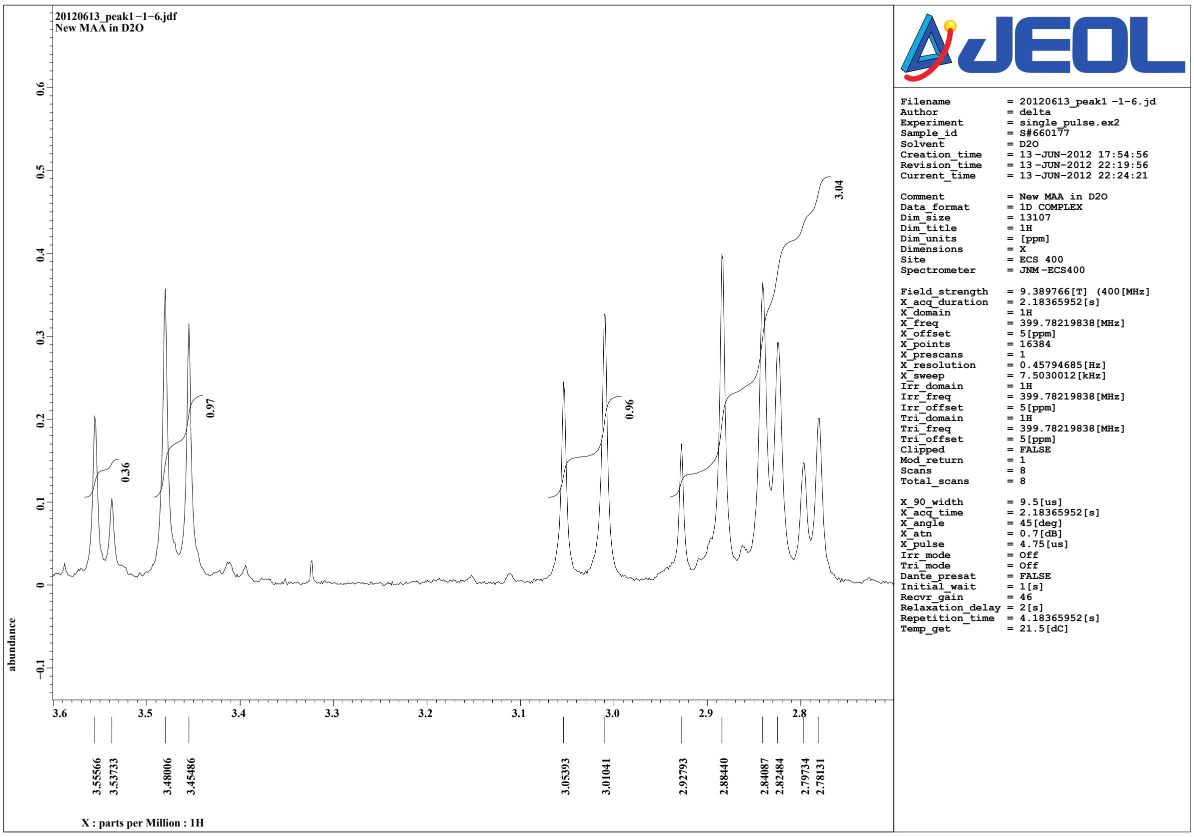

Figure S3. ^{13}C NMR spectrum of the 508-Da MAA in D_2O.

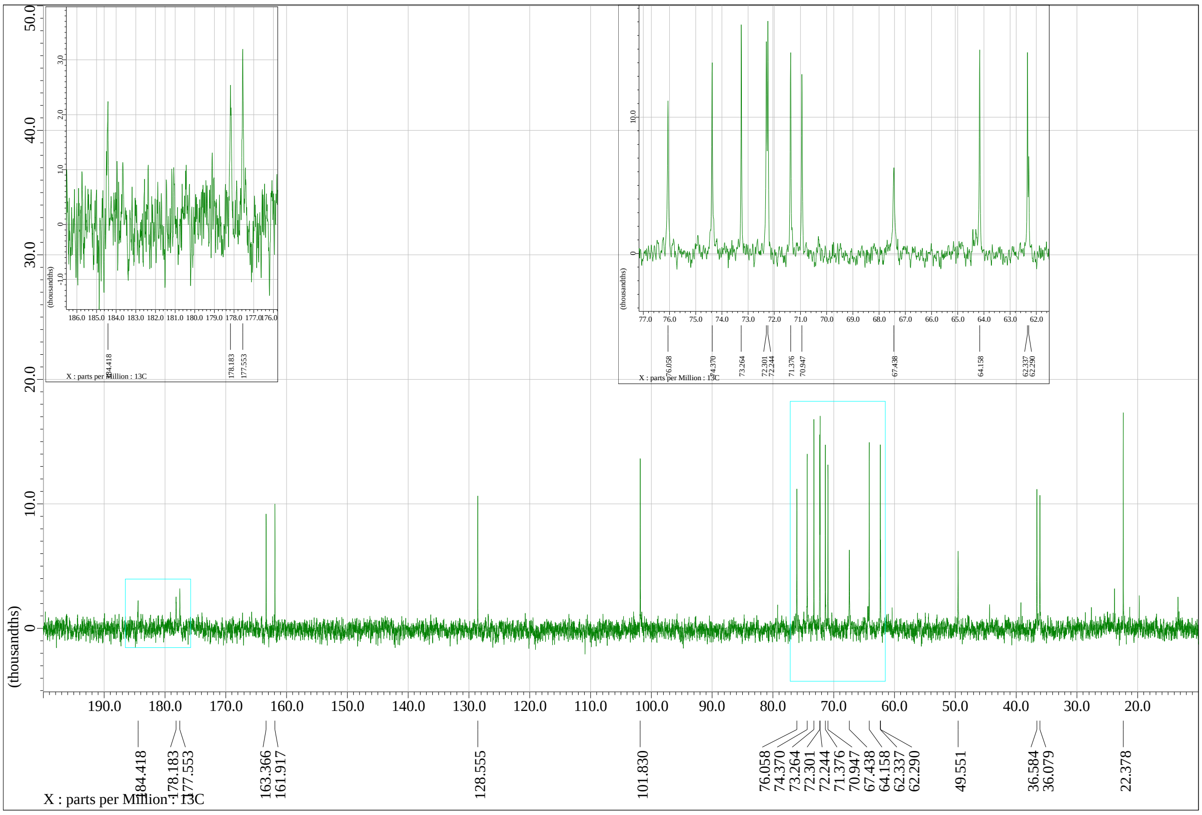

Figure S4. DEPT spectra of the 508-Da MAA in D_2O (DEPT135: upper, DEPT90: middle, 13C lower).

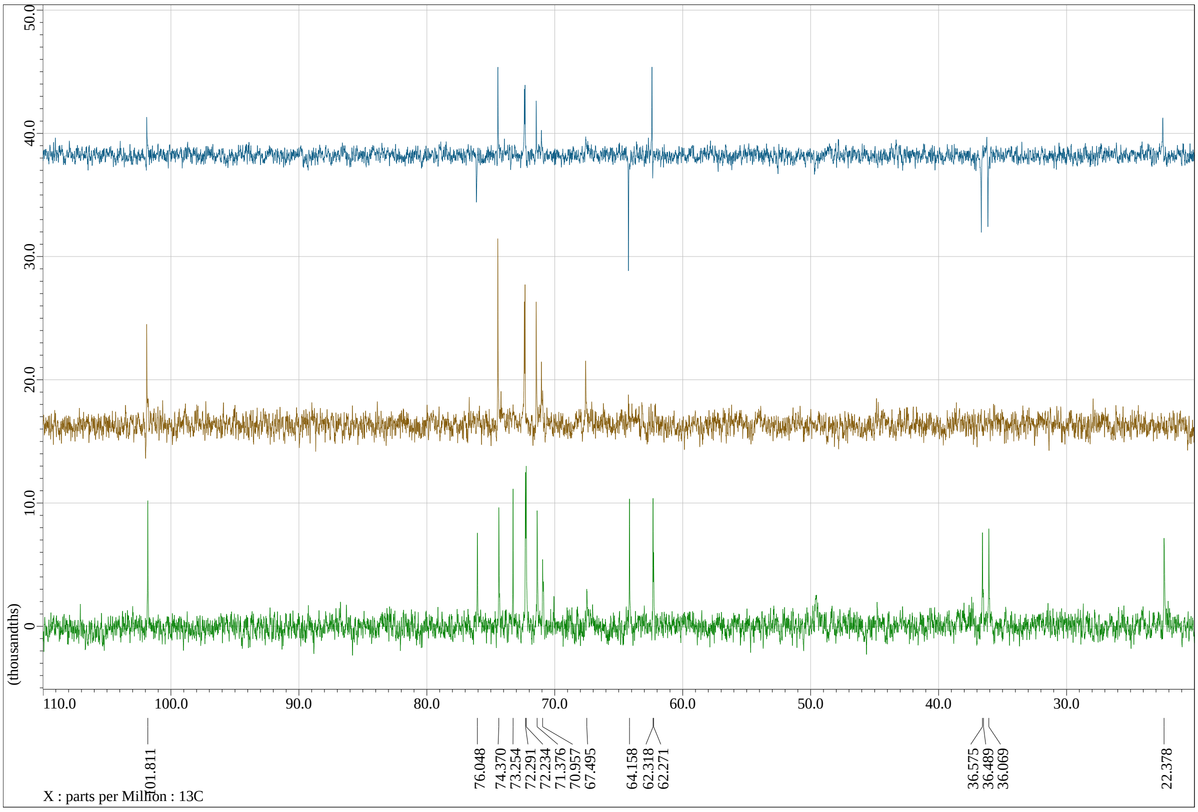

Figure S4. *Cont.*

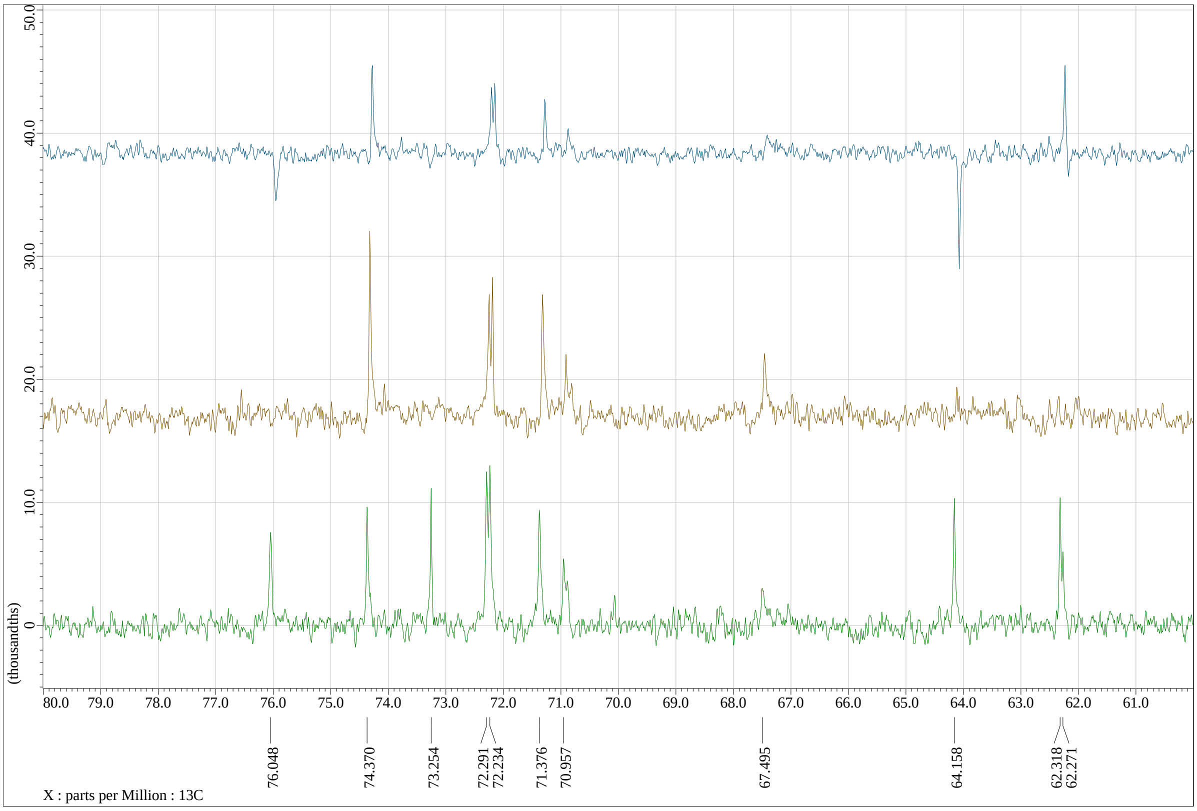

Figure S5. COSY spectra of the 508-Da MAA in D_2O.

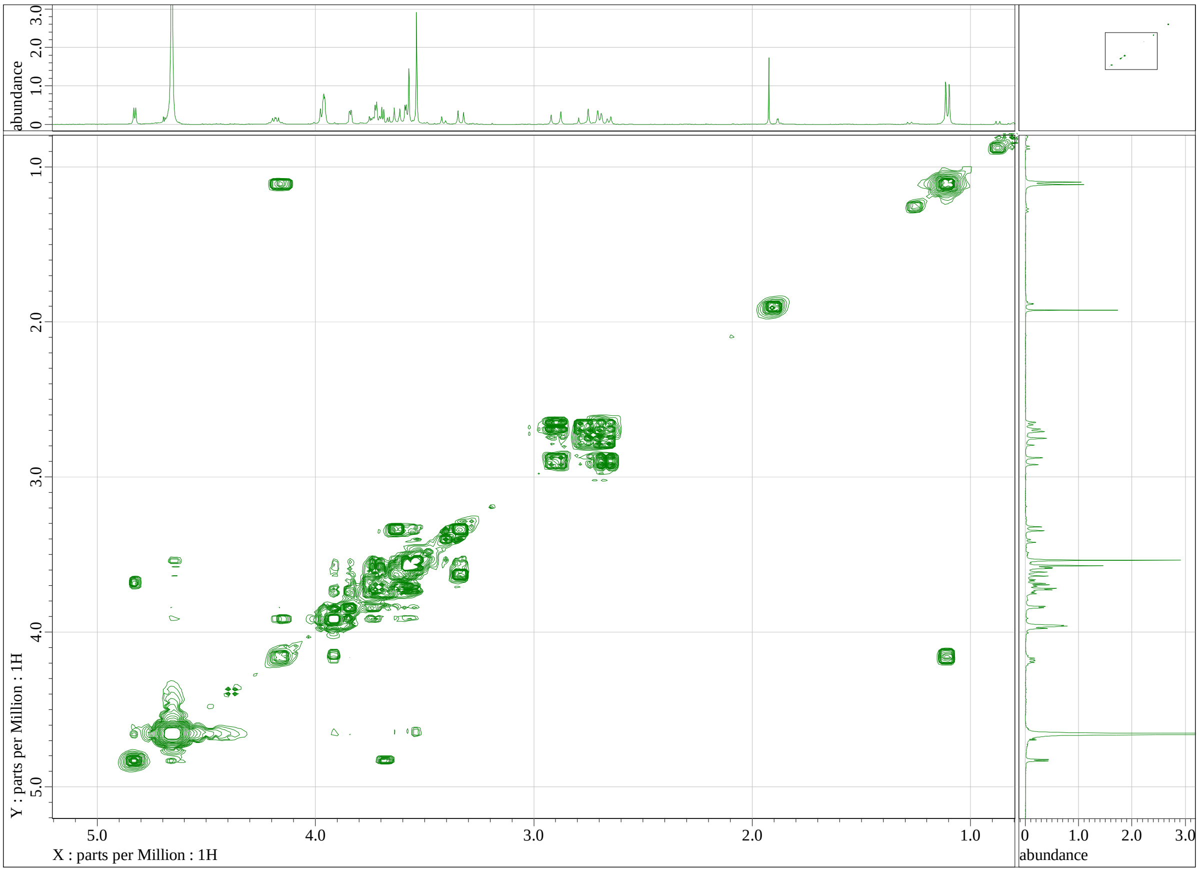

Figure S5. *Cont.*

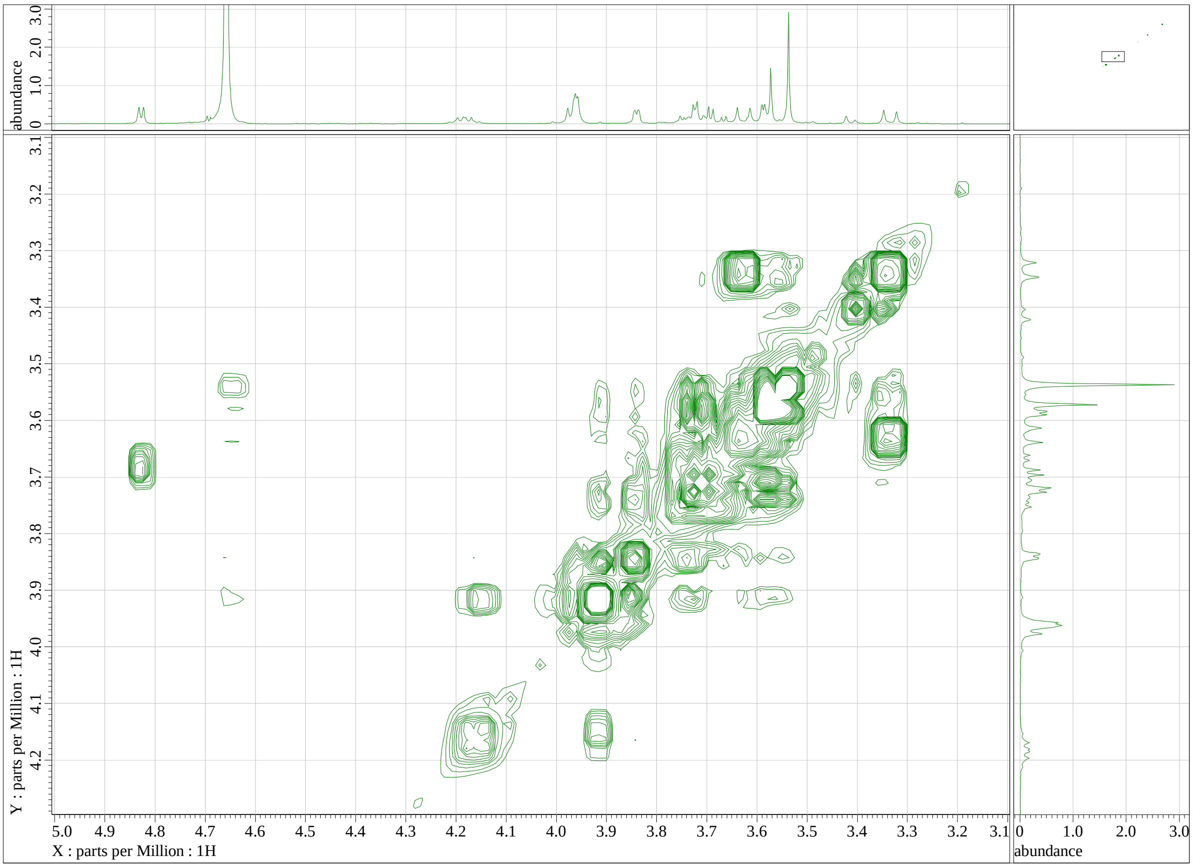

Figure S6. HMQC spectra of the 508-Da MAA in D_2O.

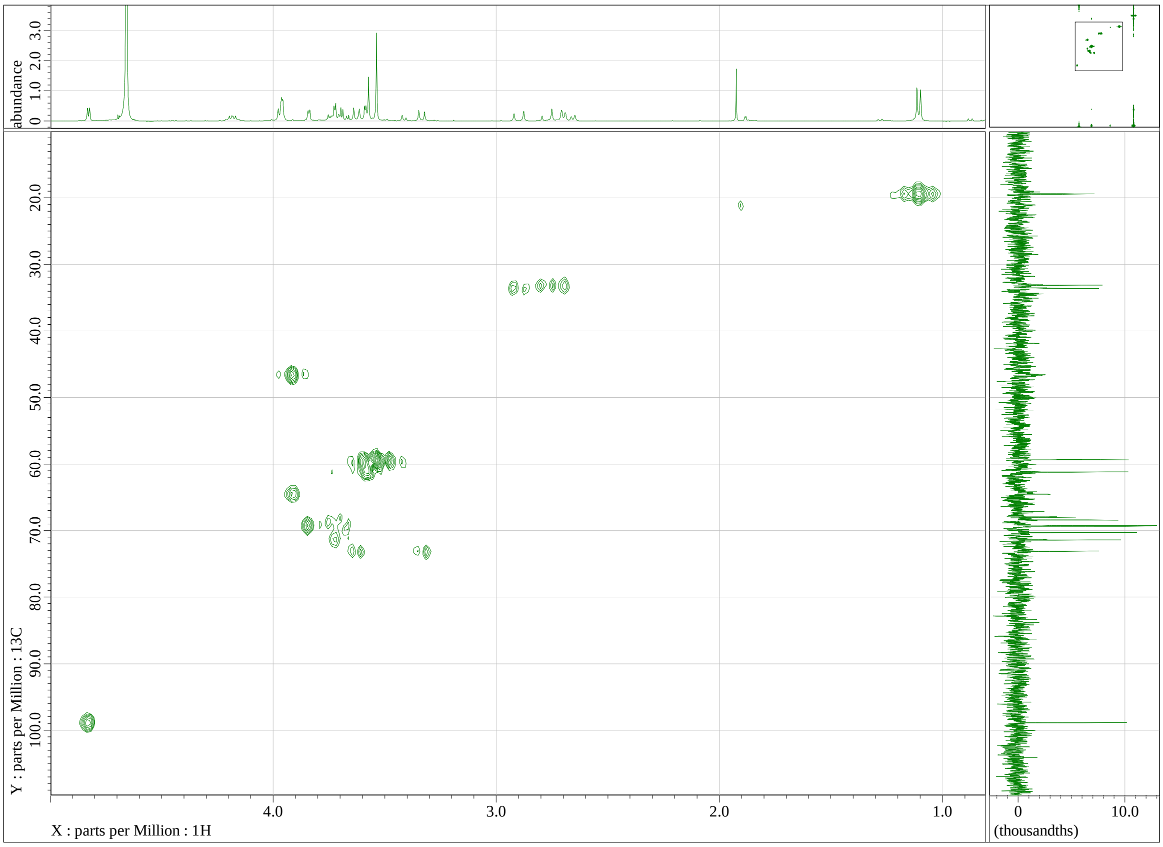

Figure S6. *Cont.*

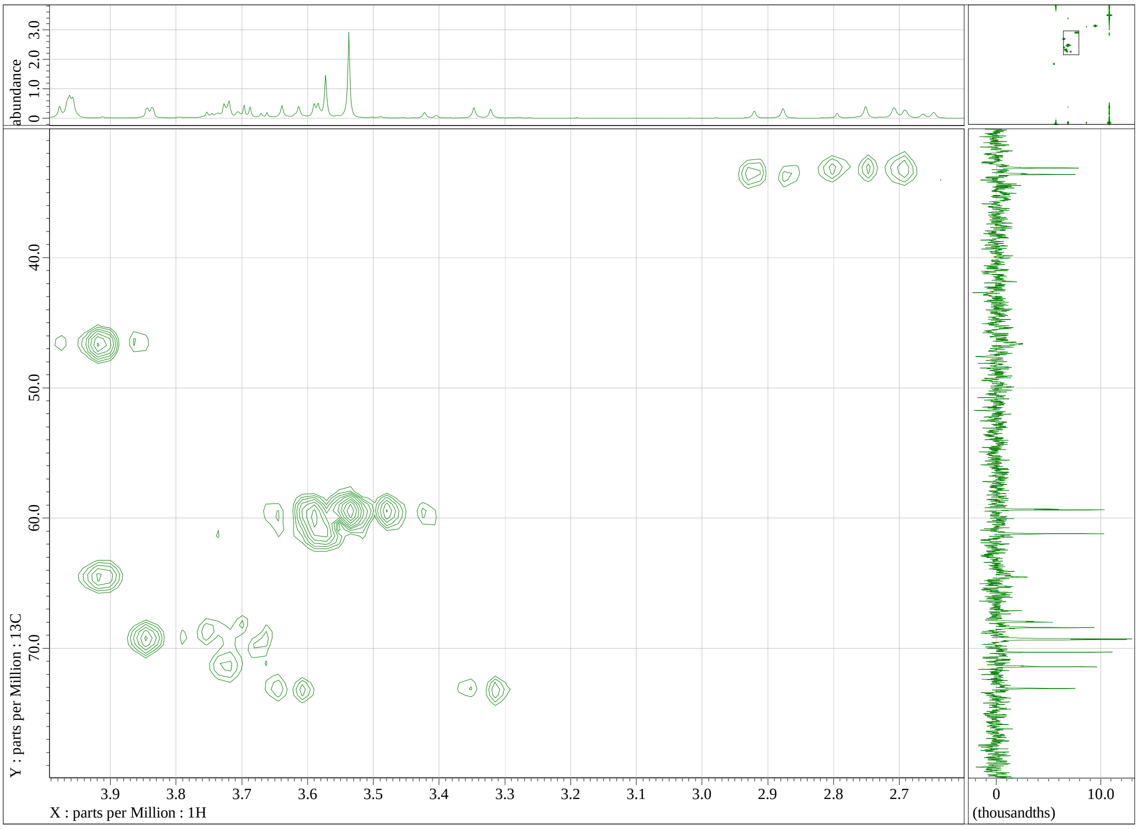

Figure S7. HMBC spectra of the 508-Da MAA in D_2O.

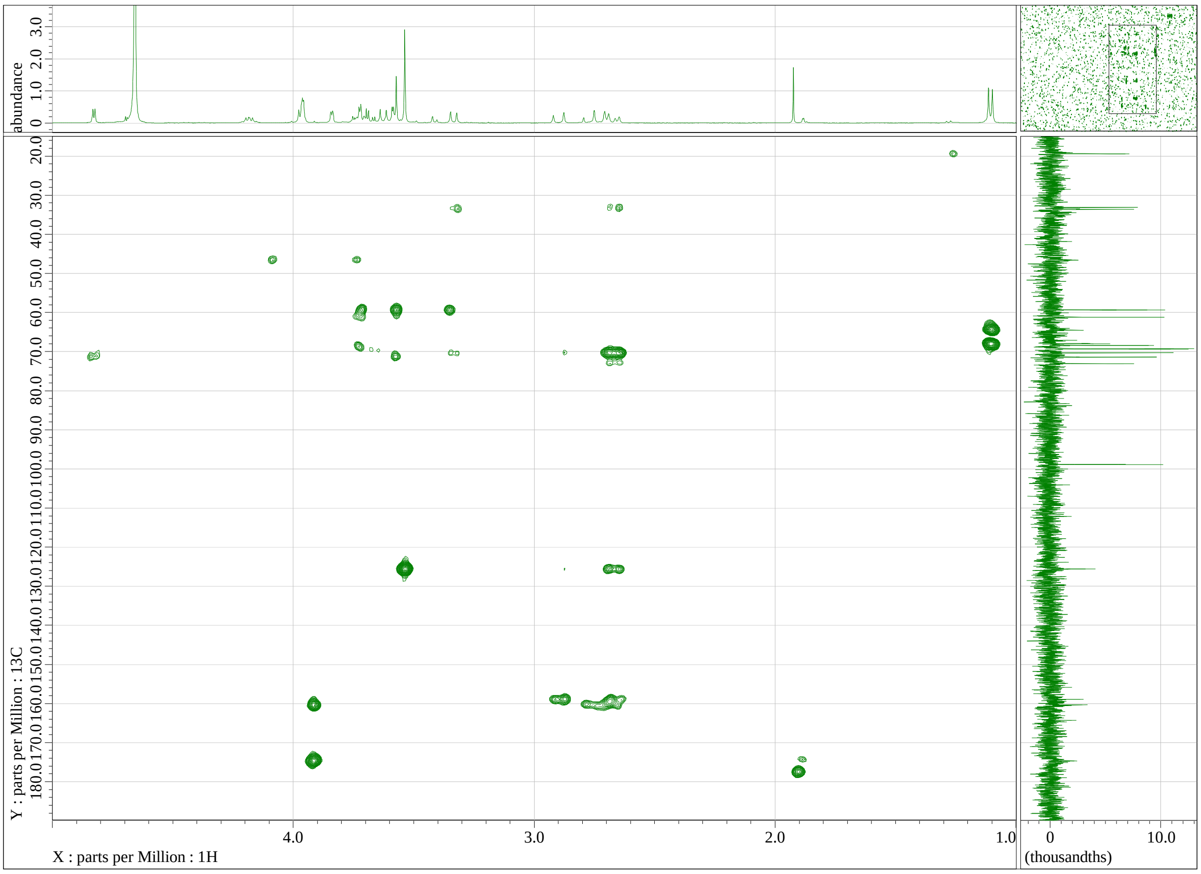

Figure S7. *Cont.*

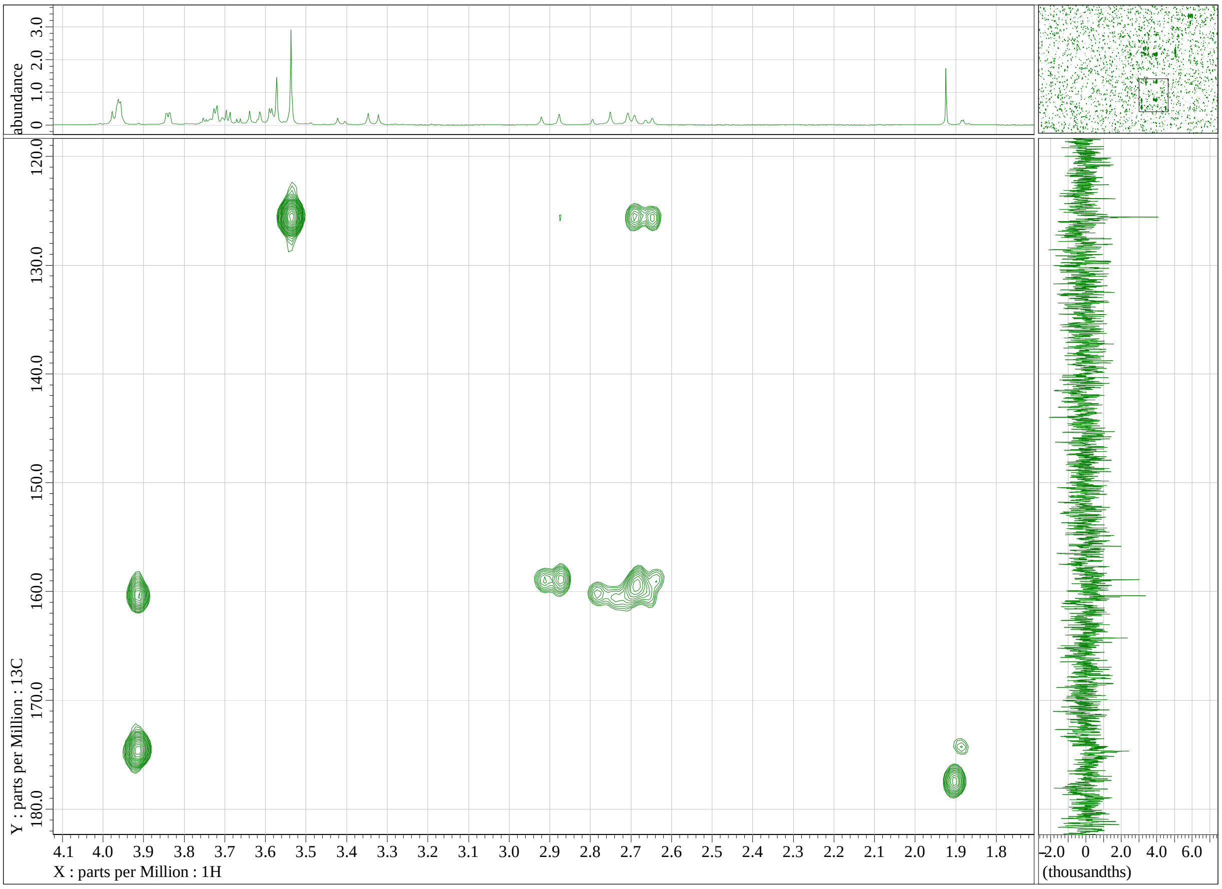

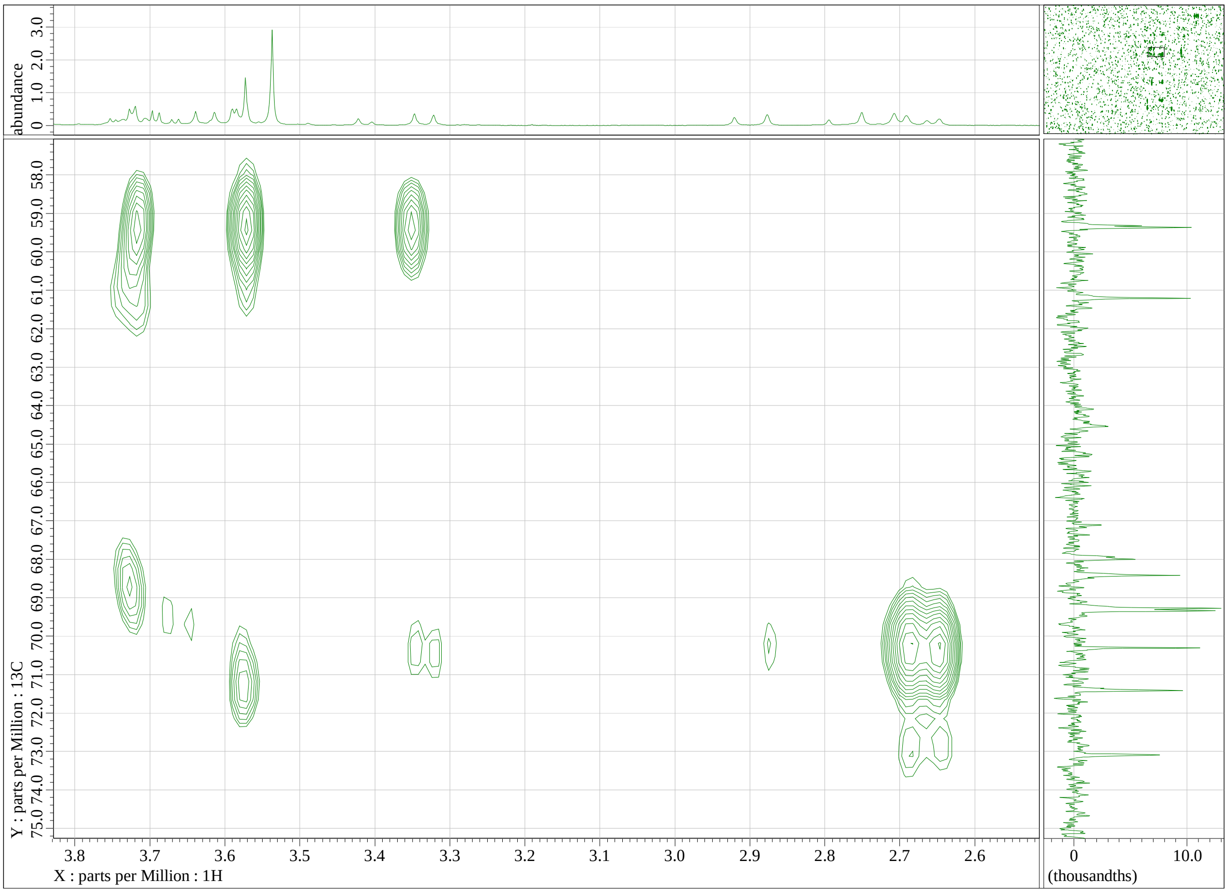

2.2. NMR Data for the 612-Da MAA

Figure S8. ^{1}H NMR spectra of the 612-Da MAA in D_2O.

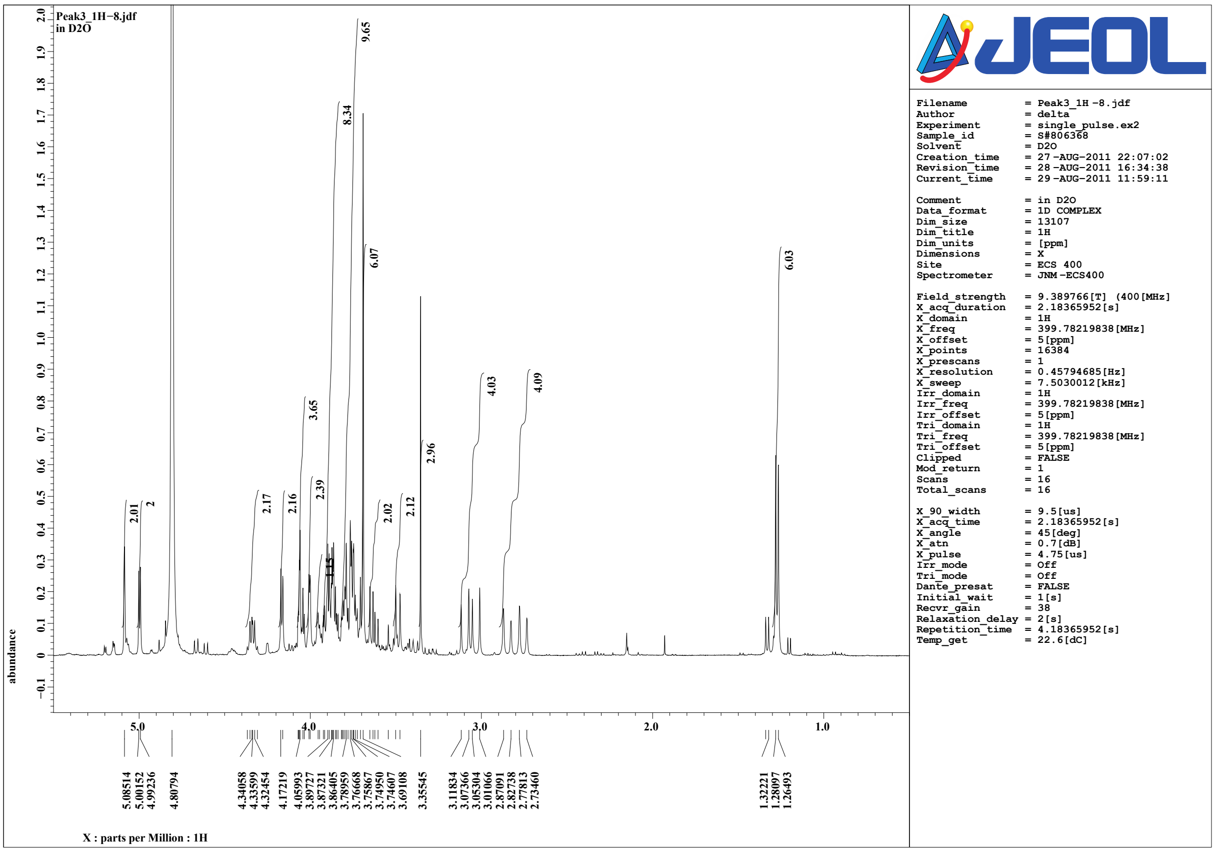

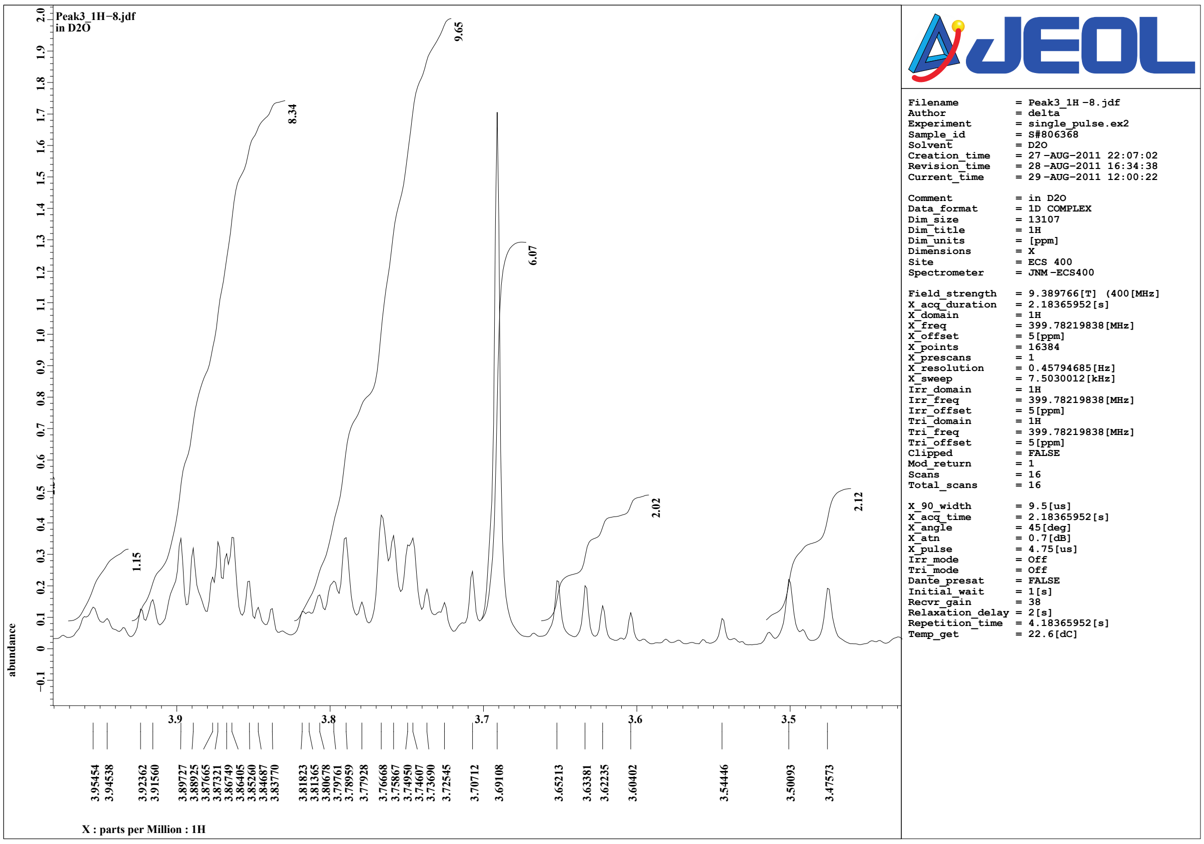

Figure S8. *Cont.*

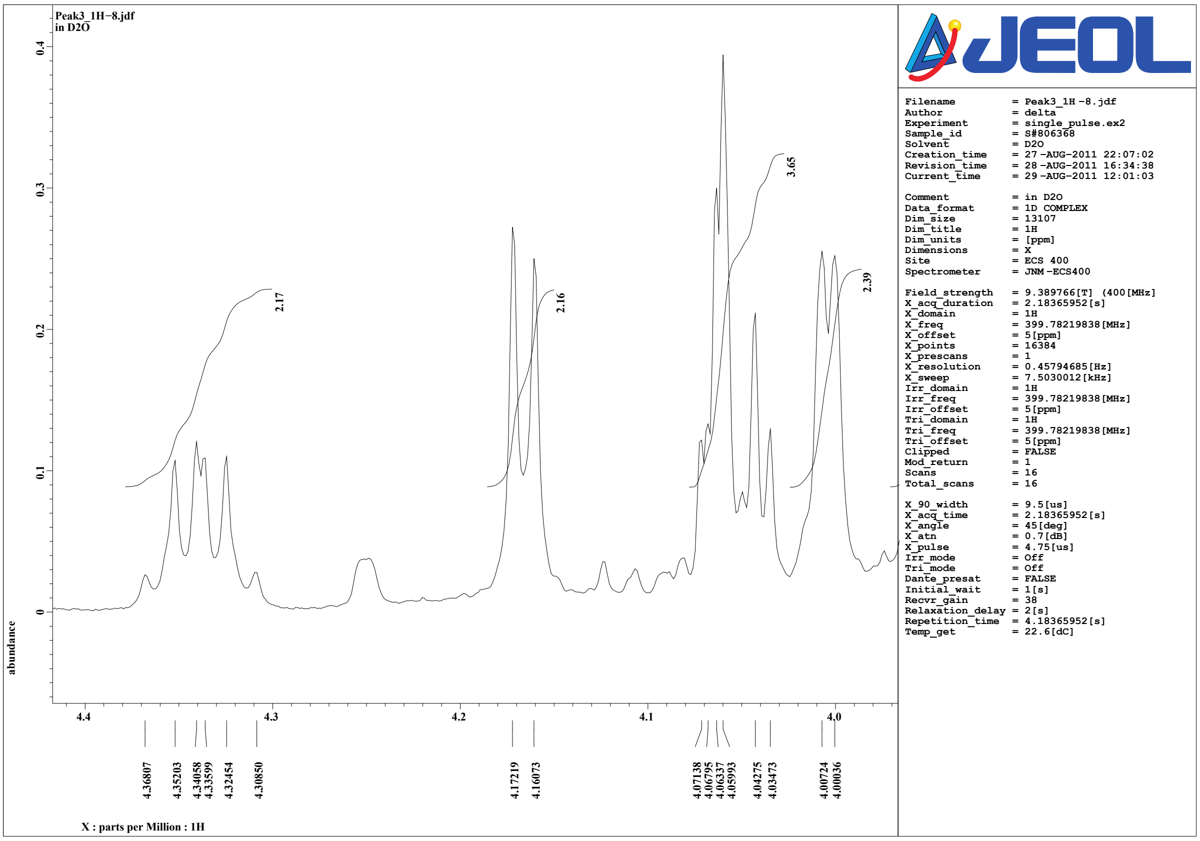

Figure S9. ^{13}C NMR spectra of the 612-Da MAA in D_2O.

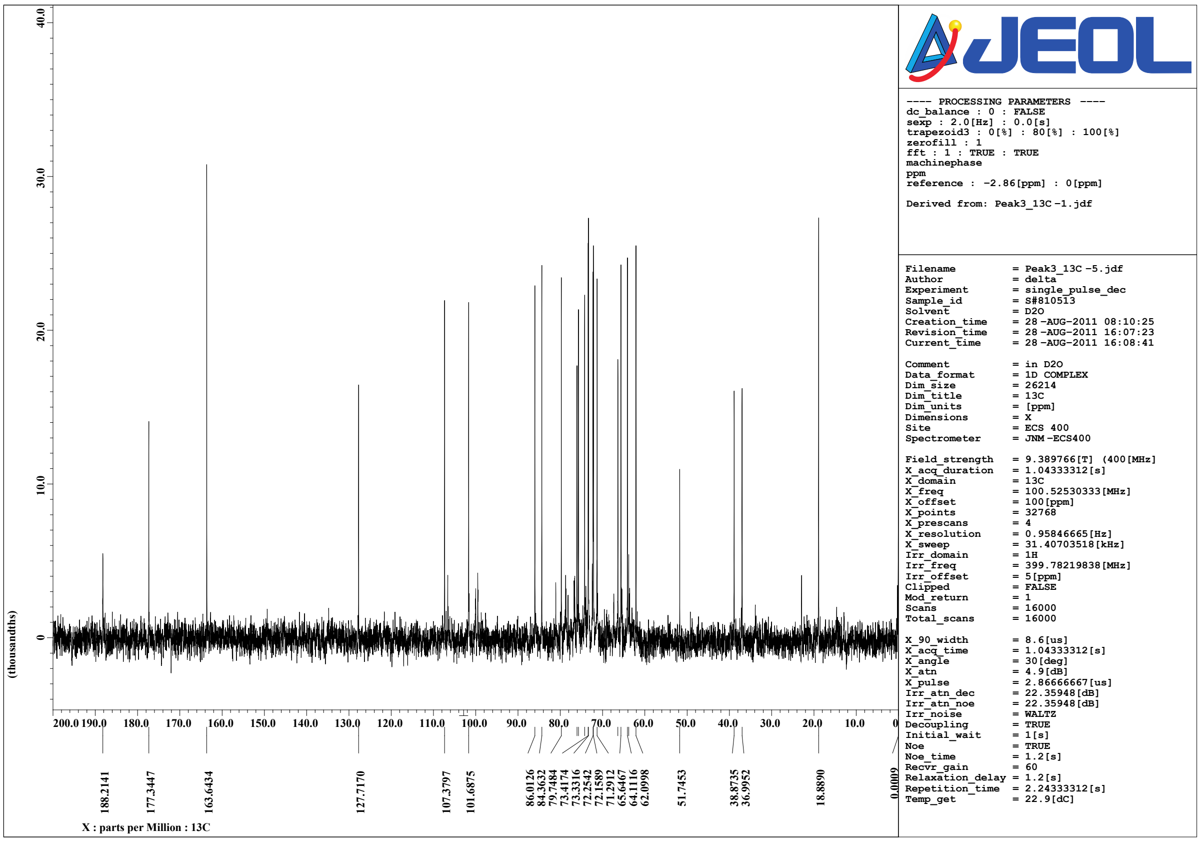

Figure S9. *Cont.*

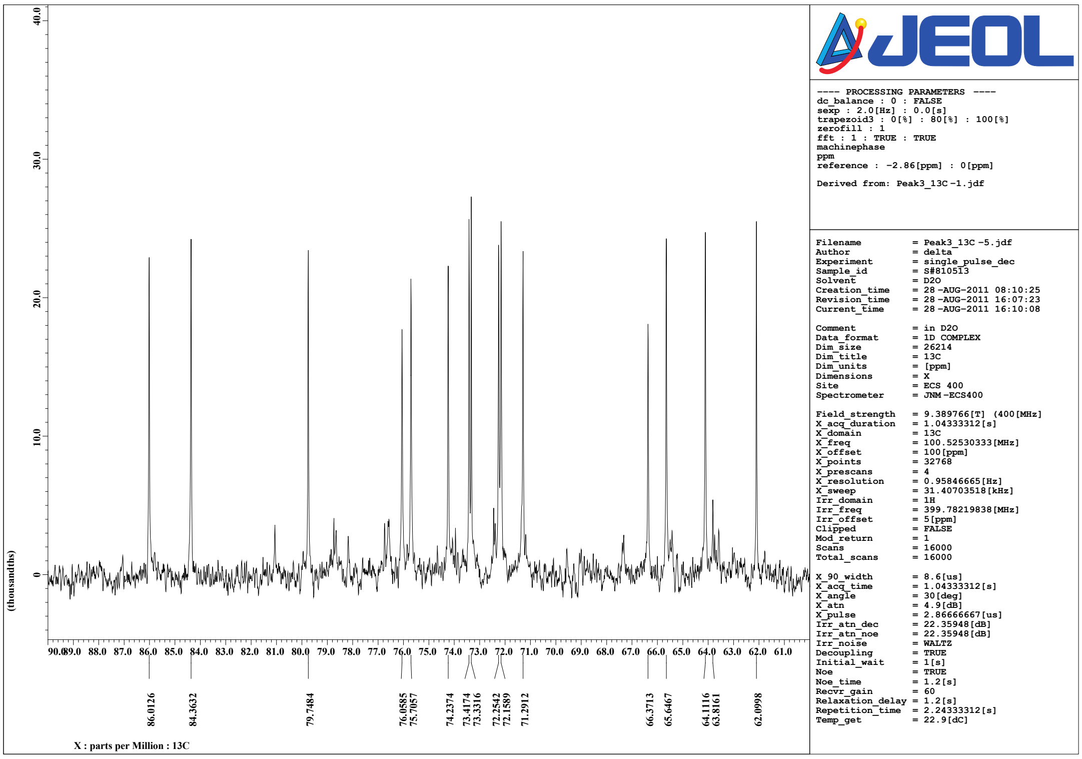

Figure S10. High resolution ^{13}C NMR spectrum of the 612-Da MAA in D_2O at around 160 ppm. Focusing on the narrow X-range at around 160 ppm, ^{13}C NMR spectrum was recorded with a high resolution to separate the two signals from C1 and C3. Because these chemical shift values were not adjusted by referring internal standard at 0 ppm, the chemical shift values recorded here were different from those in the ^{13}C NMR spectrum shown in Figure S9.

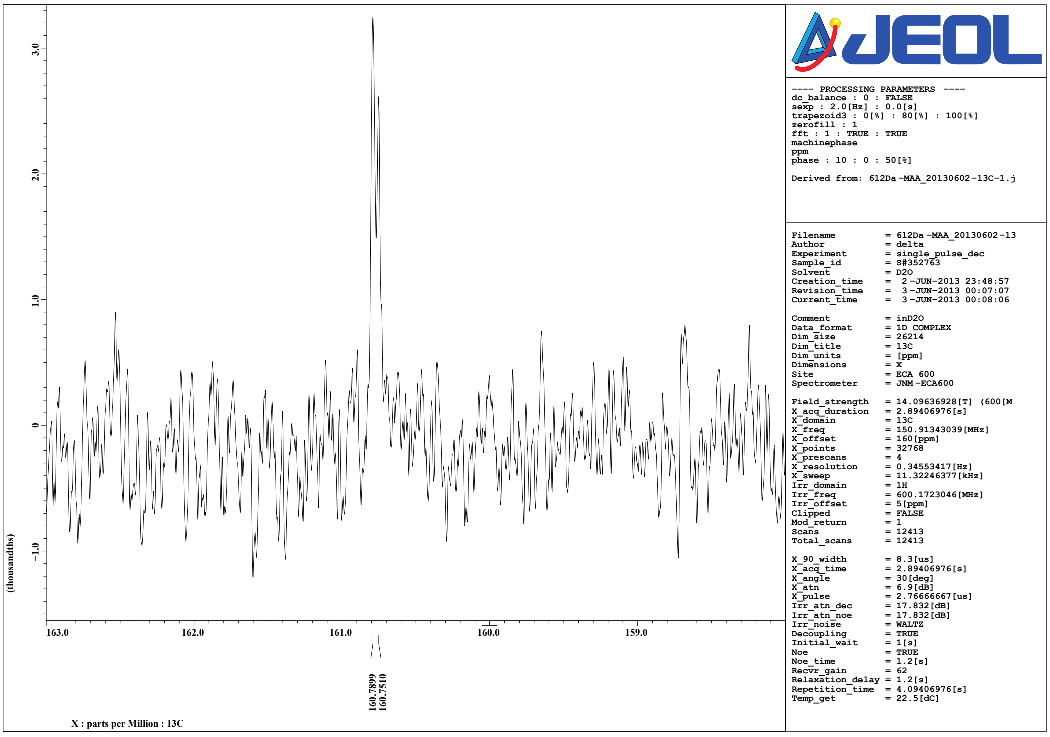

Figure S11. DEPT90 (upper) and ^{13}C (lower) NMR spectra of the 612-Da MAA in D_2O.

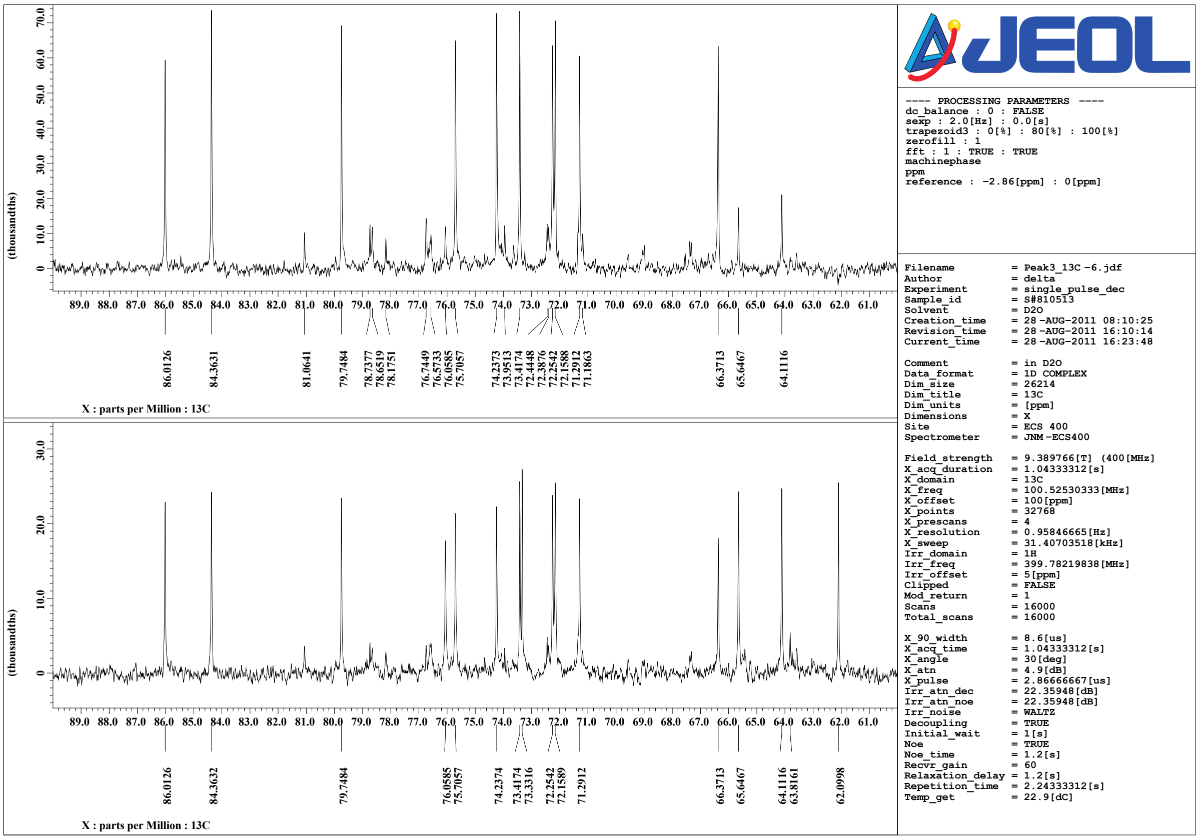

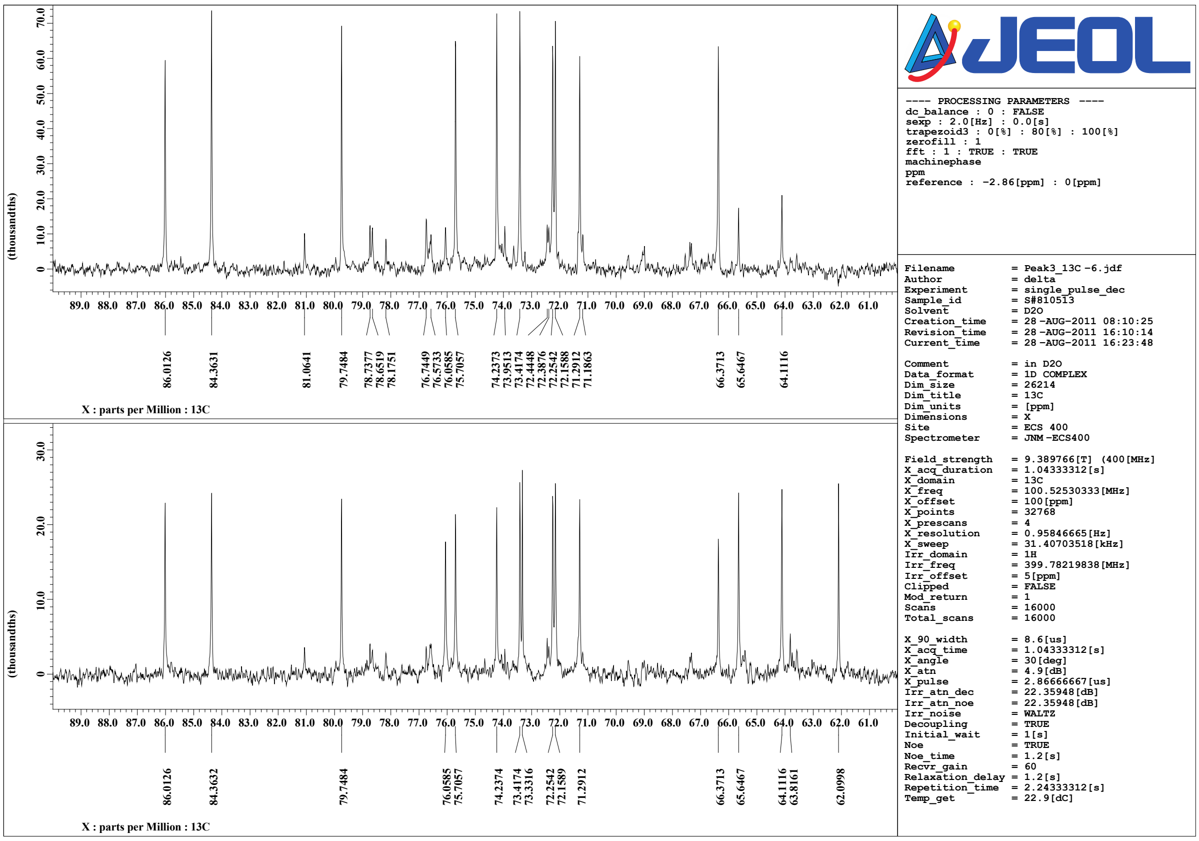

Figure S12. DEPT135 (upper) and ^{13}C (lower) NMR spectra of the 612-Da MAA in D_2O.

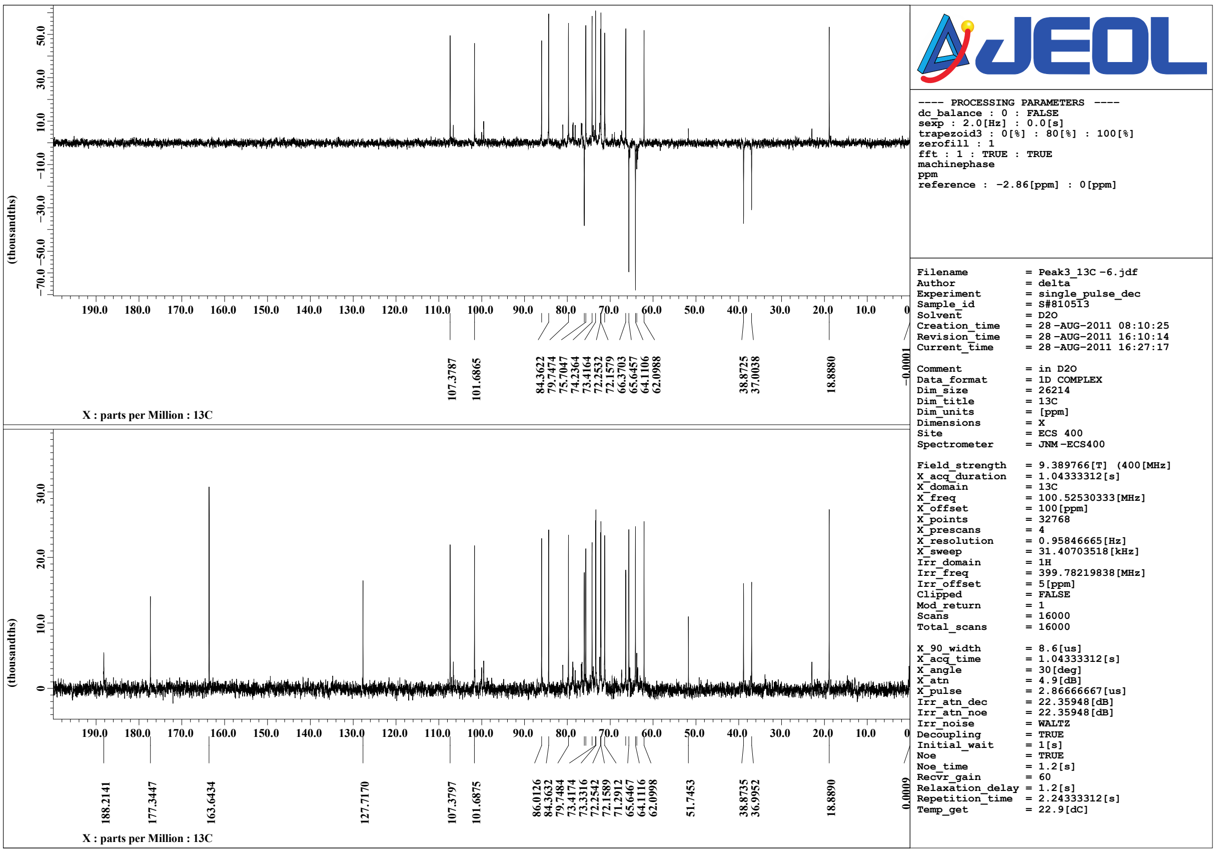

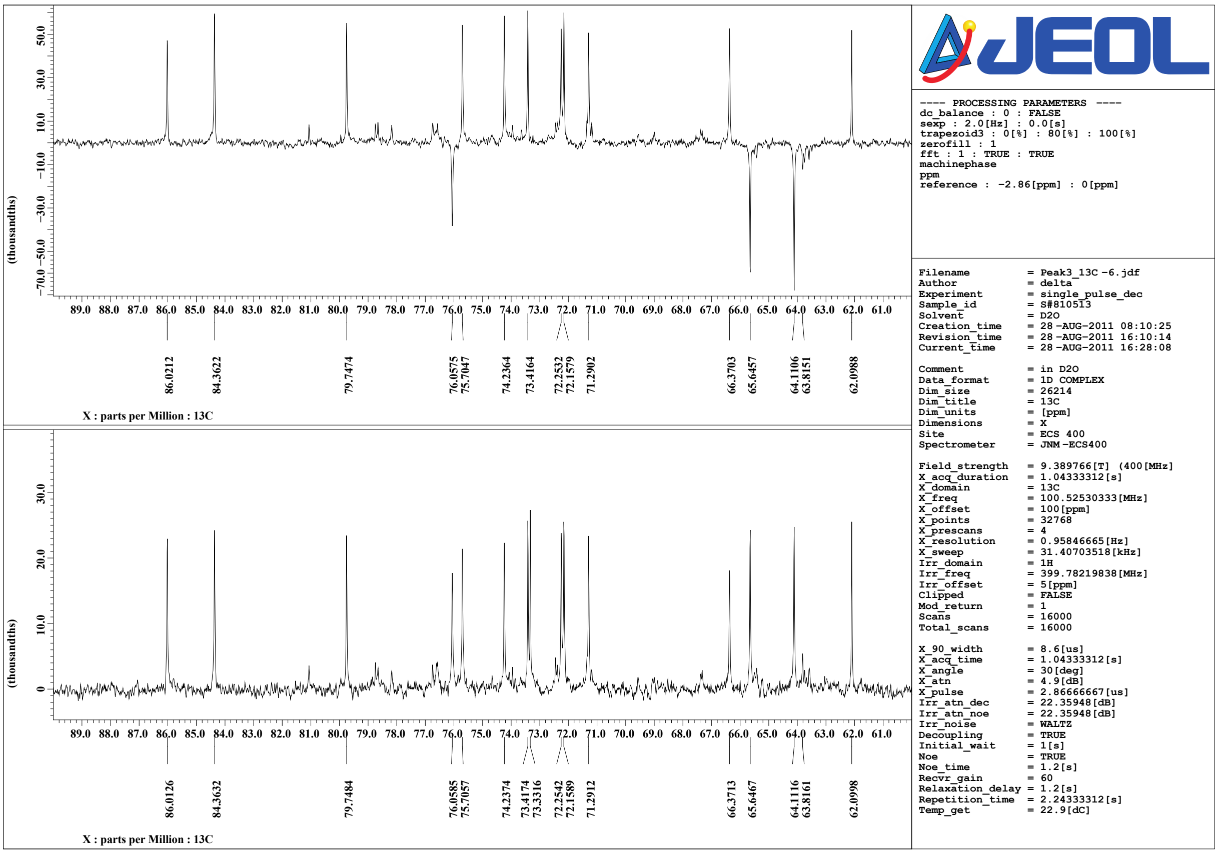

Figure S13. COSY spectra of the 612-Da MAA in D_2O.

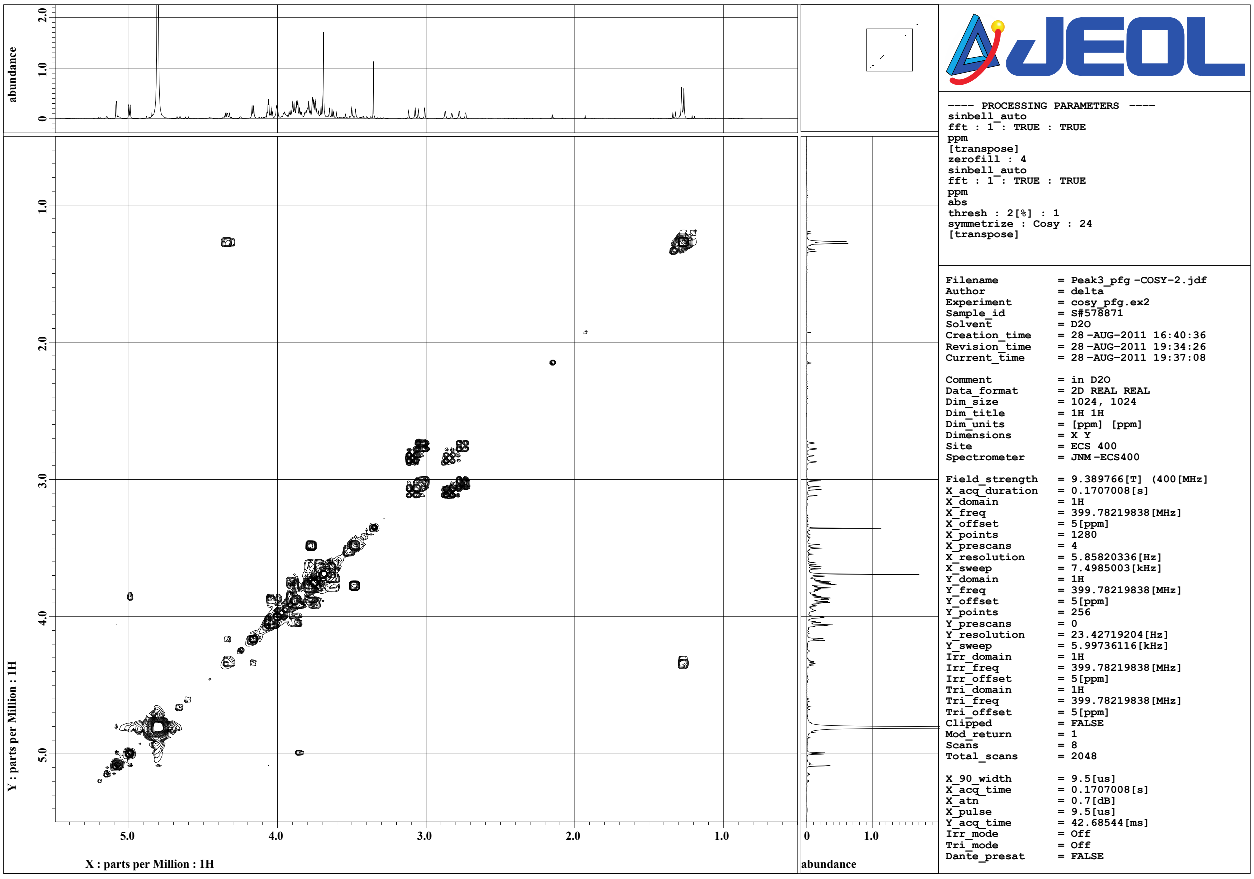

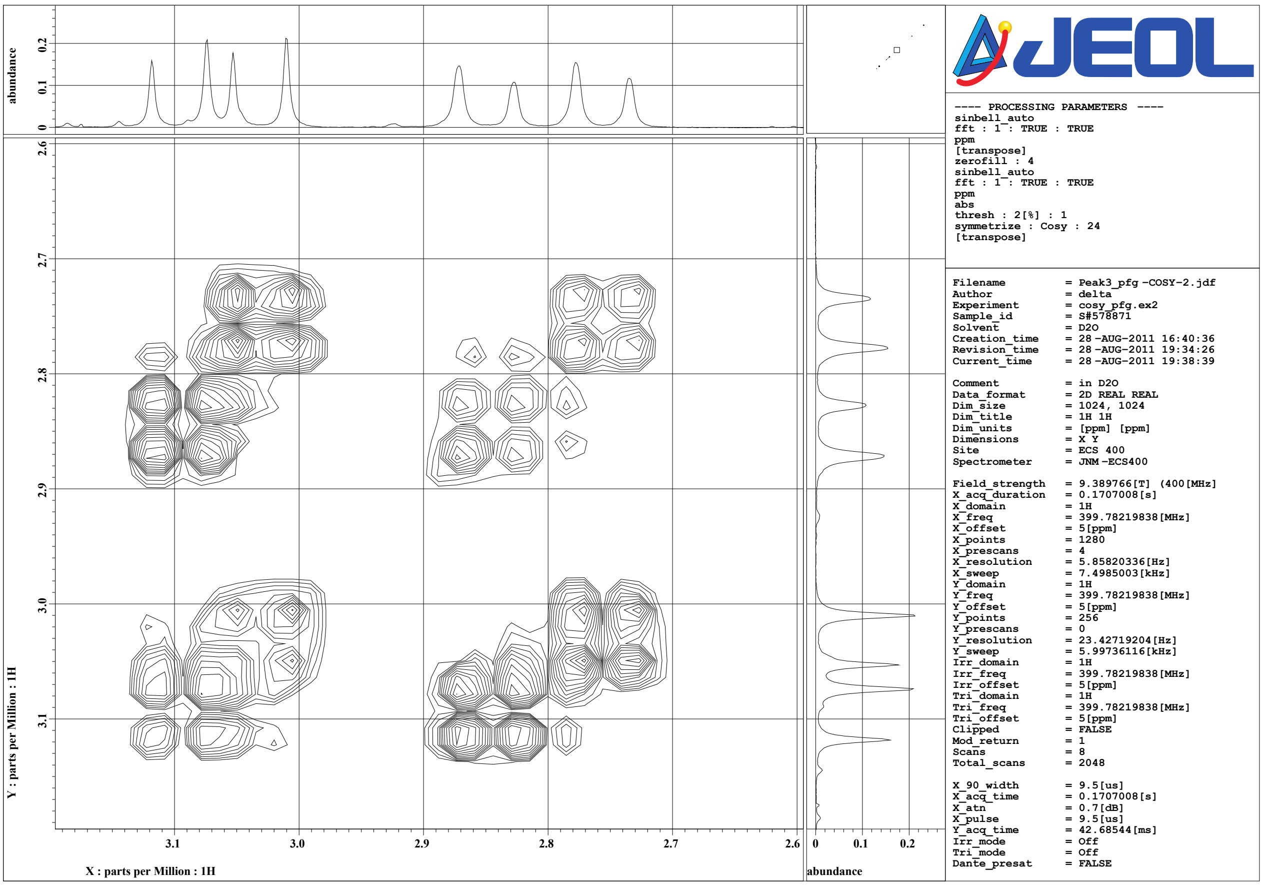

Figure S13. *Cont.*

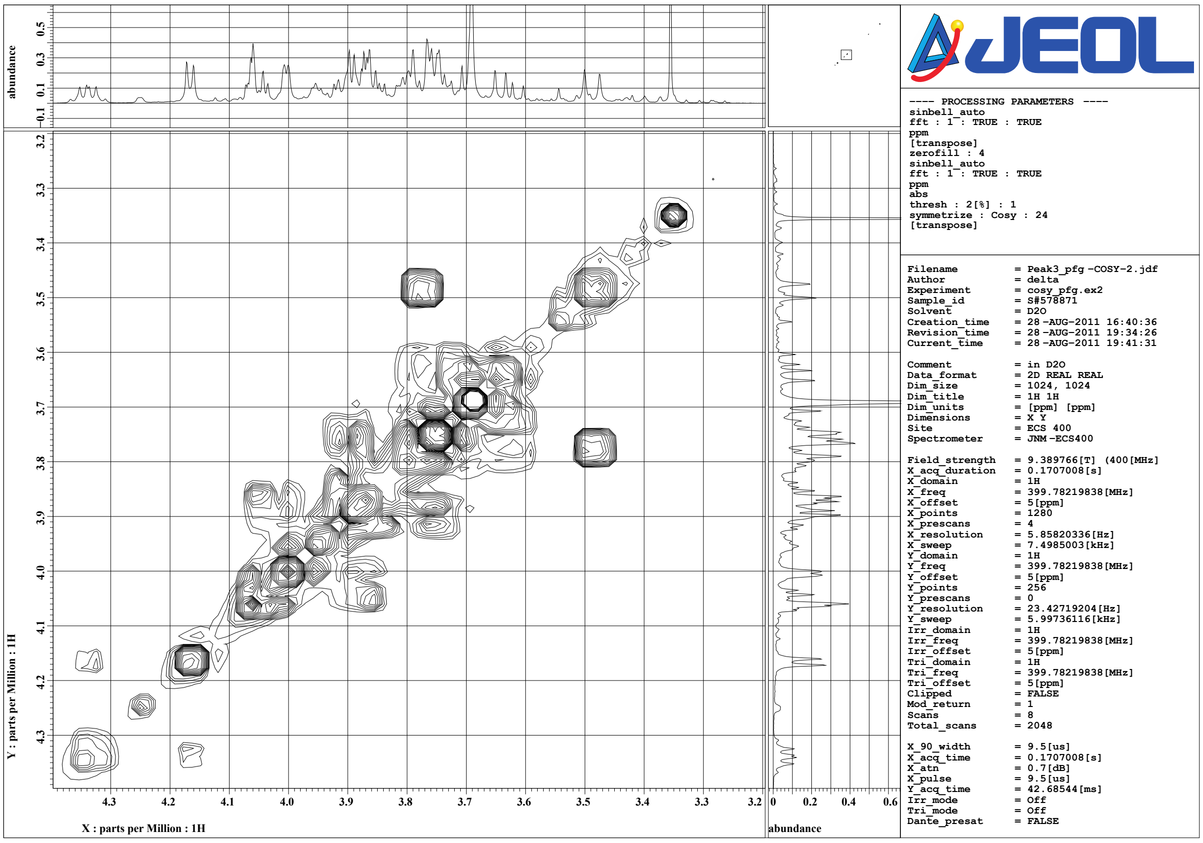

Figure S14. HMQC spectra of the 612-Da MAA in D_2O.

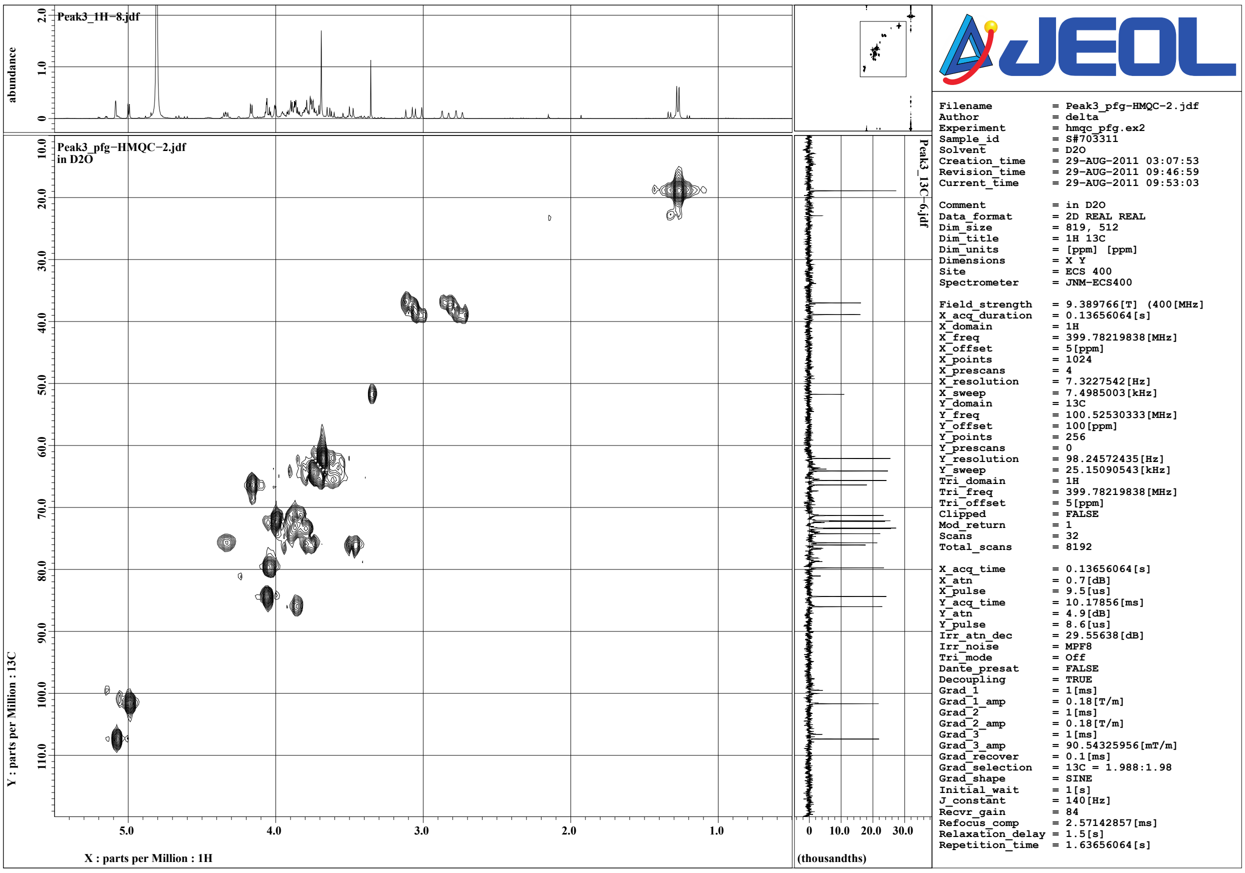

Figure S14. *Cont.*

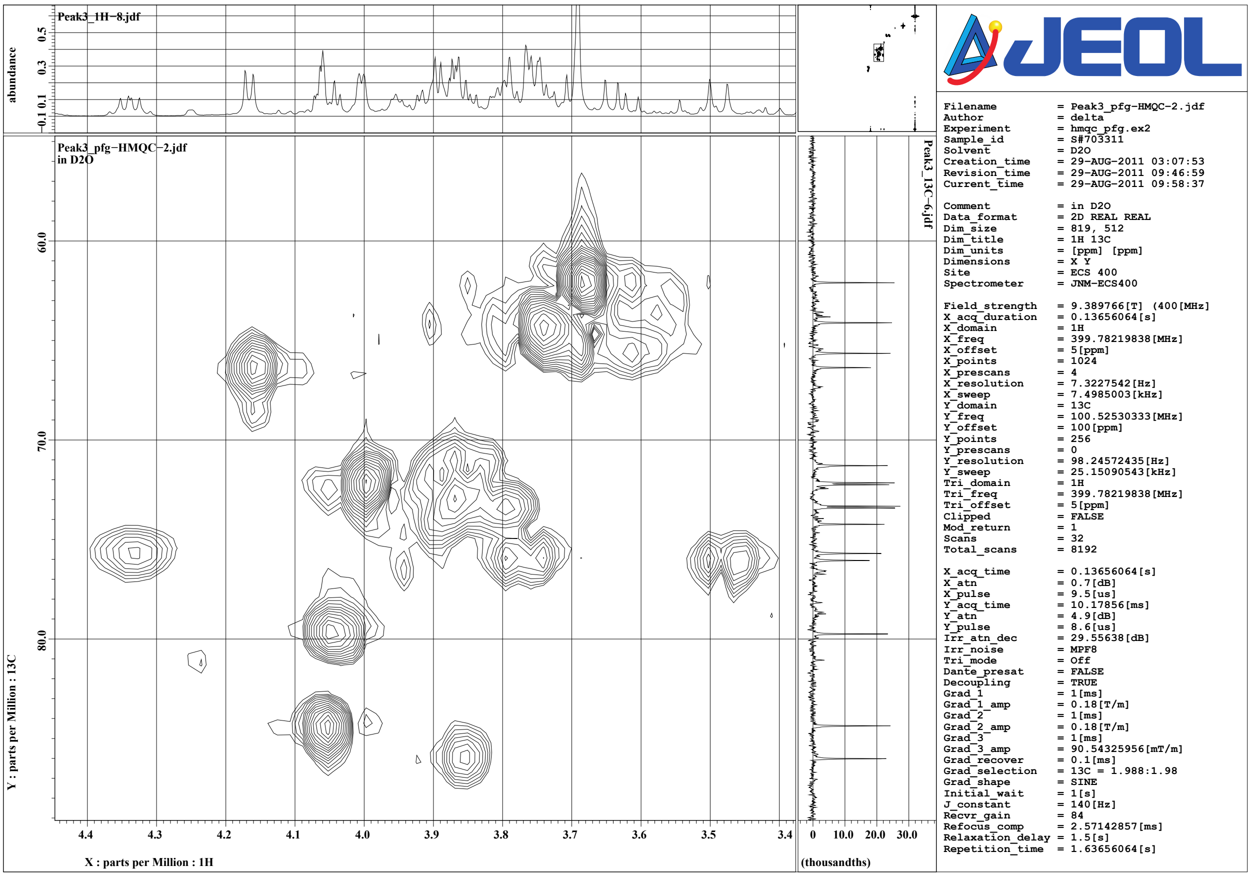

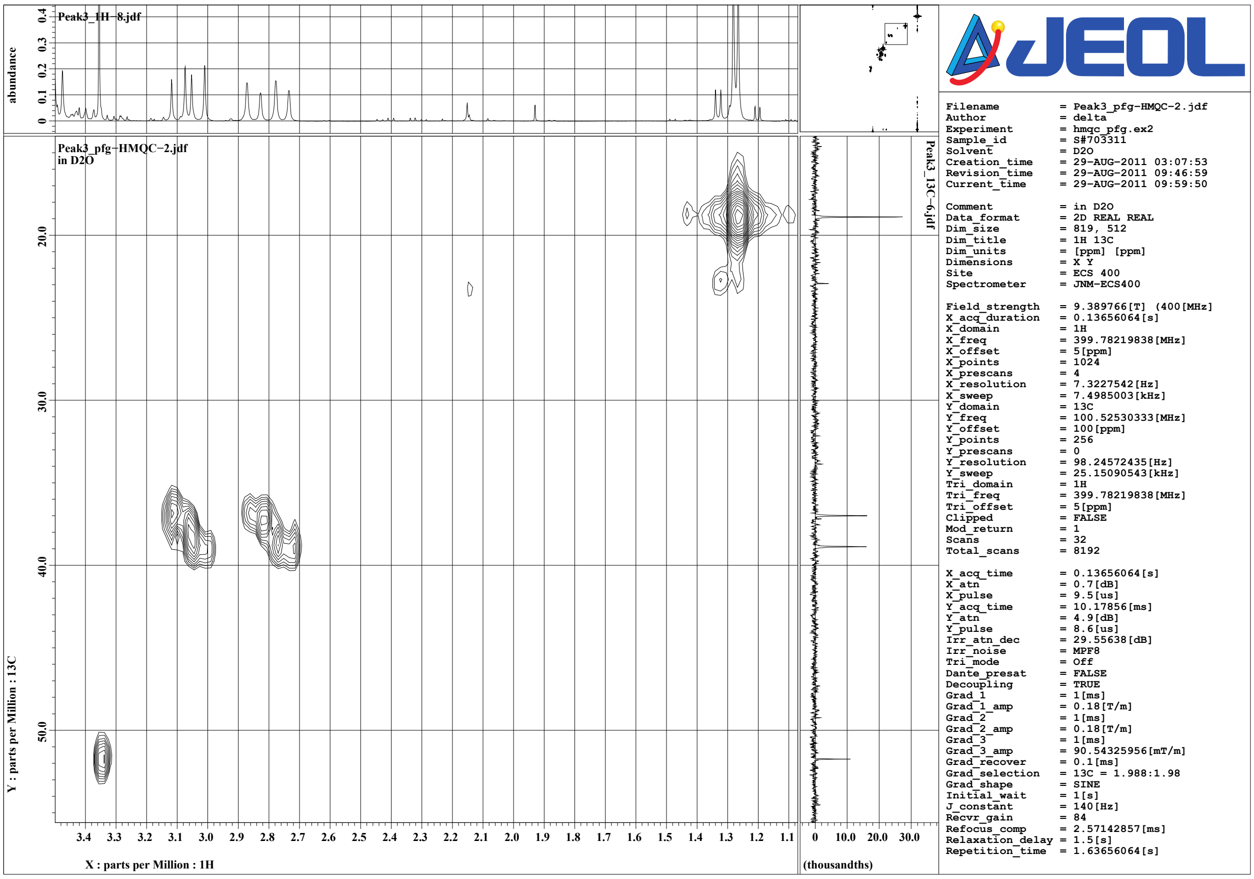

Figure S15. HMBC spectra of the 612-Da MAA in D_2O.

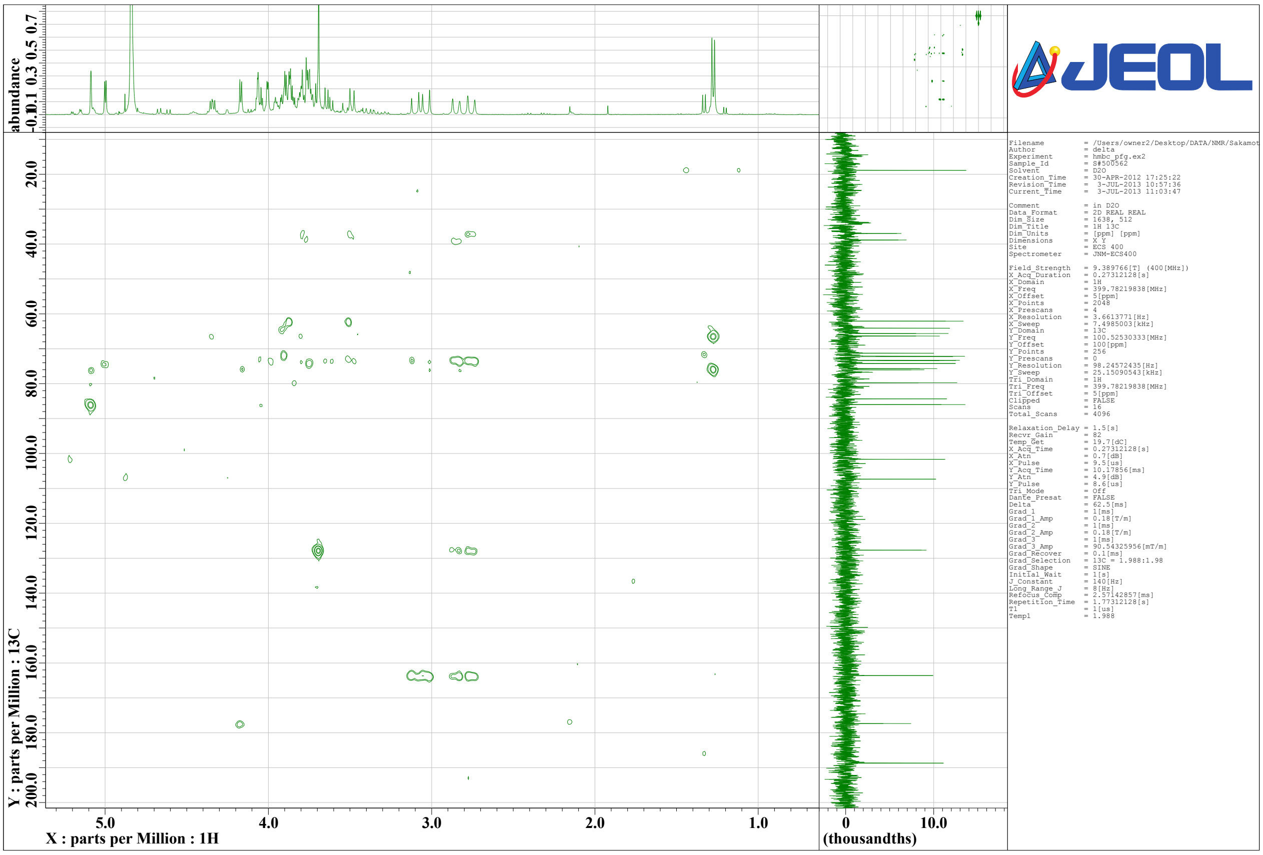

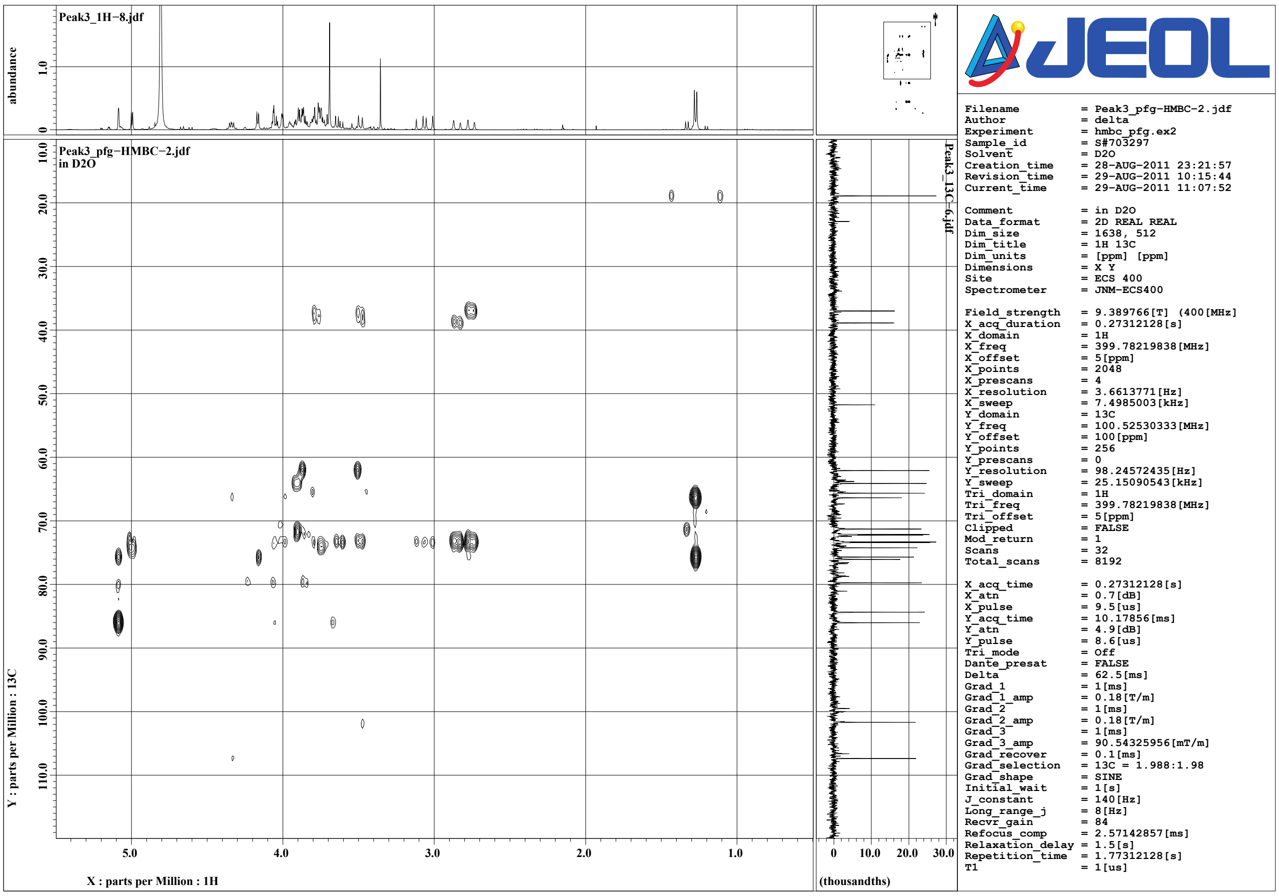

Figure S15. *Cont.*

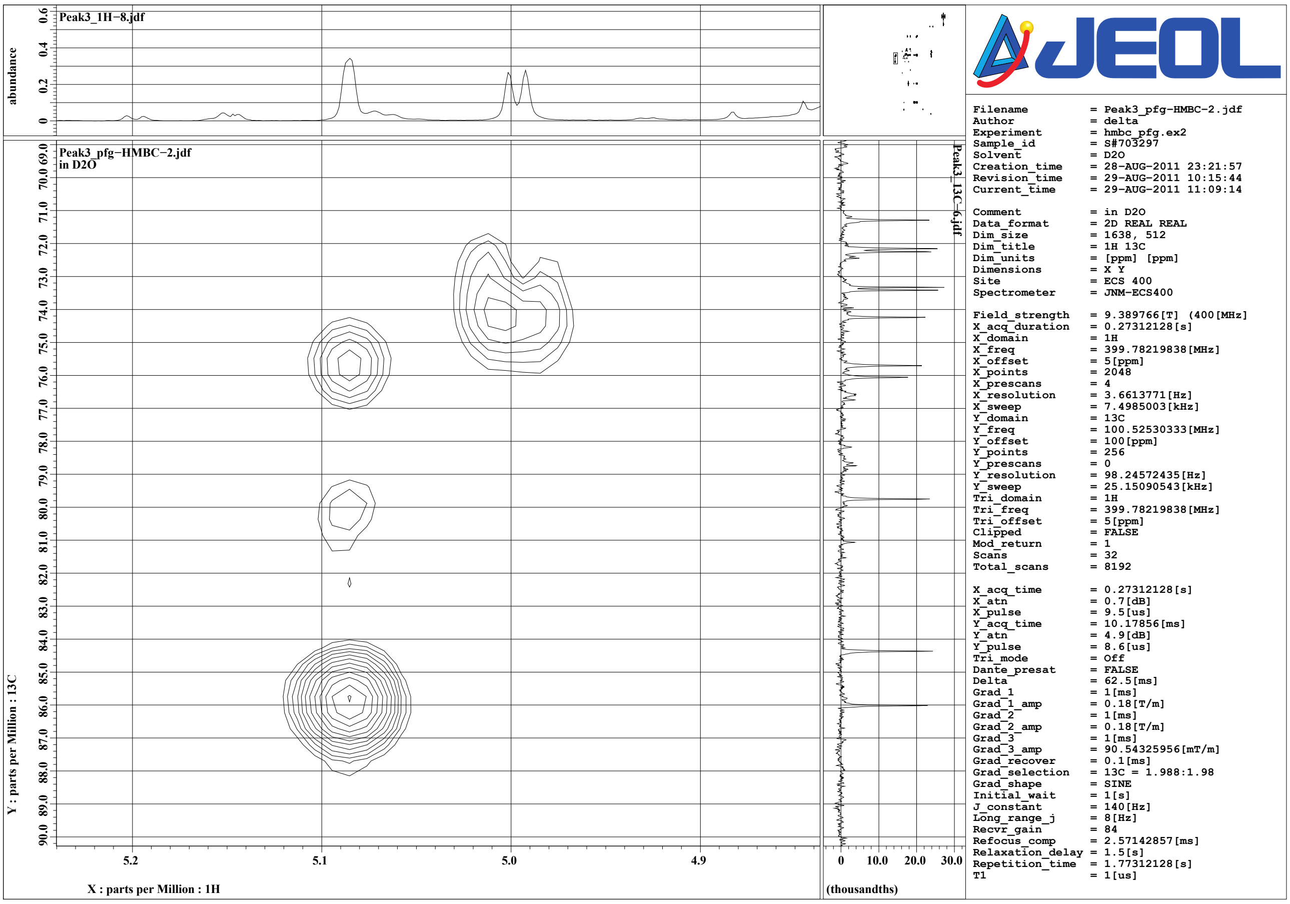

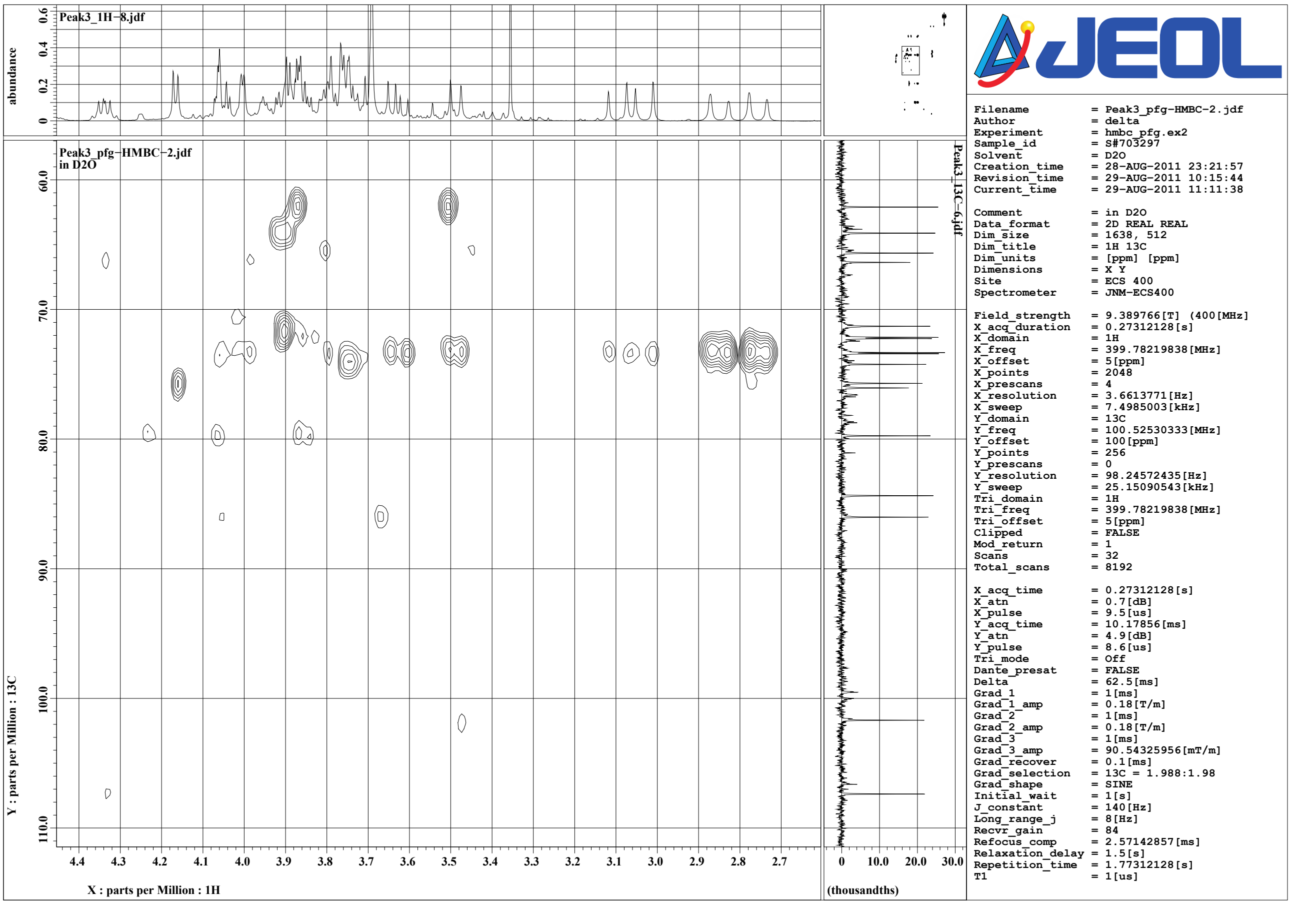

Figure S15. *Cont.*

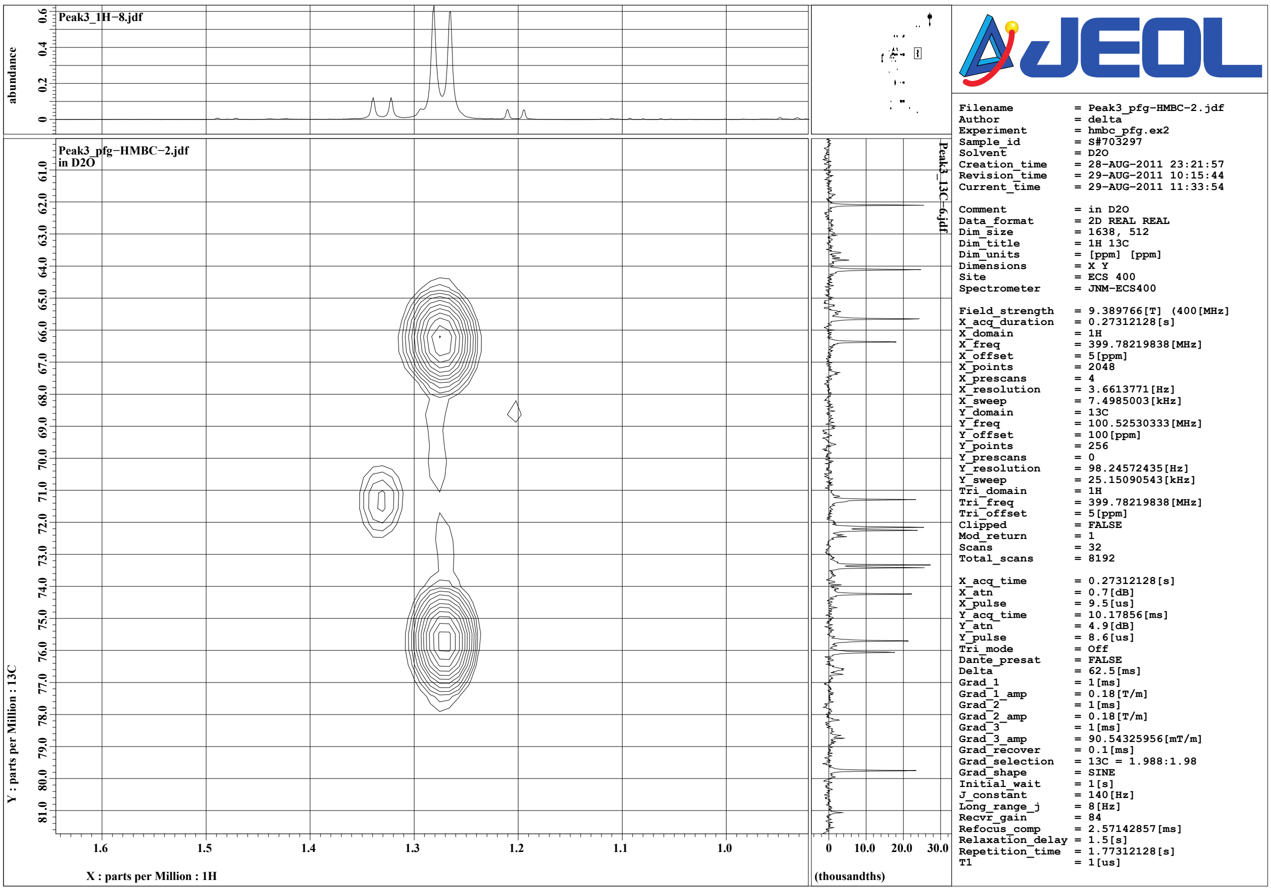

Supplement: Supplementary File 1 — Supporting Information (PDF, 2994 KB) [file marinedrugs-11-03124-s001.pdf]
